# Ψ-Footprinting approach for the identification of protein synthesis inhibitor producers

**DOI:** 10.1093/nargab/lqac055

**Published:** 2022-07-15

**Authors:** Franziska Handel, Andreas Kulik, Katharina W Wex, Anne Berscheid, Julian S Saur, Anika Winkler, Daniel Wibberg, Jörn Kalinowski, Heike Brötz-Oesterhelt, Yvonne Mast

**Affiliations:** Department of Microbiology/Biotechnology, Interfaculty Institute of Microbiology and Infection Medicine, Faculty of Science, University of Tübingen, Auf der Morgenstelle 28, 72076 Tübingen, Germany; German Center for Infection Research (DZIF), Partner Site Tübingen, Tübingen, Germany; Department of Microbiology/Biotechnology, Interfaculty Institute of Microbiology and Infection Medicine, Faculty of Science, University of Tübingen, Auf der Morgenstelle 28, 72076 Tübingen, Germany; Department of Microbial Bioactive Compounds; Interfaculty Institute of Microbiology and Infection Medicine, University of Tübingen; Tübingen, Baden-Württemberg 72076, Germany; Department of Microbial Bioactive Compounds; Interfaculty Institute of Microbiology and Infection Medicine, University of Tübingen; Tübingen, Baden-Württemberg 72076, Germany; German Center for Infection Research (DZIF), Partner Site Tübingen, Tübingen, Germany; Department of Microbial Bioactive Compounds; Interfaculty Institute of Microbiology and Infection Medicine, University of Tübingen; Tübingen, Baden-Württemberg 72076, Germany; German Center for Infection Research (DZIF), Partner Site Tübingen, Tübingen, Germany; Biomolecular Chemistry, Institute of Organic Chemistry, University of Tübingen, Tübingen, Baden-Württemberg 72076, Germany; Center for Biotechnology (CeBiTec), Bielefeld University, Universitätsstraße 27, 33615 Bielefeld, Germany; Center for Biotechnology (CeBiTec), Bielefeld University, Universitätsstraße 27, 33615 Bielefeld, Germany; Center for Biotechnology (CeBiTec), Bielefeld University, Universitätsstraße 27, 33615 Bielefeld, Germany; Department of Microbial Bioactive Compounds; Interfaculty Institute of Microbiology and Infection Medicine, University of Tübingen; Tübingen, Baden-Württemberg 72076, Germany; German Center for Infection Research (DZIF), Partner Site Tübingen, Tübingen, Germany; Cluster of Excellence Controlling Microbes to Fight Infection, Germany; Department of Microbiology/Biotechnology, Interfaculty Institute of Microbiology and Infection Medicine, Faculty of Science, University of Tübingen, Auf der Morgenstelle 28, 72076 Tübingen, Germany; German Center for Infection Research (DZIF), Partner Site Tübingen, Tübingen, Germany; Department Bioresources for Bioeconomy and Health Research, Leibniz Institute DSMZ - German Collection of Microorganisms and Cell Cultures, Inhoffenstraße 7B, 38124 Braunschweig, Germany; Technical University Braunschweig, Department of Microbiology, Rebenring 56, 38106 Braunschweig, Germany

## Abstract

Today, one of the biggest challenges in antibiotic research is a targeted prioritization of natural compound producer strains and an efficient dereplication process to avoid undesired rediscovery of already known substances. Thereby, genome sequence-driven mining strategies are often superior to wet-lab experiments because they are generally faster and less resource-intensive. In the current study, we report on the development of a novel *in silico* screening approach to evaluate the genetic potential of bacterial strains to produce protein synthesis inhibitors (PSI), which was termed the protein synthesis inhibitor ('psi’) target gene footprinting approach = Ψ-footprinting. The strategy is based on the occurrence of protein synthesis associated self-resistance genes in genome sequences of natural compound producers. The screening approach was applied to 406 genome sequences of actinomycetes strains from the DSMZ strain collection, resulting in the prioritization of 15 potential PSI producer strains. For twelve of them, extract samples showed protein synthesis inhibitory properties in *in vitro* transcription/translation assays. For four strains, namely *Saccharopolyspora flava* DSM 44771, *Micromonospora aurantiaca* DSM 43813, *Nocardioides albertanoniae* DSM 25218, and *Geodermatophilus nigrescens* DSM 45408, the protein synthesis inhibitory substance amicoumacin was identified by HPLC-MS analysis, which proved the functionality of the *in silico* screening approach.

## Graphical Abstract

**Graphical Abstract:**
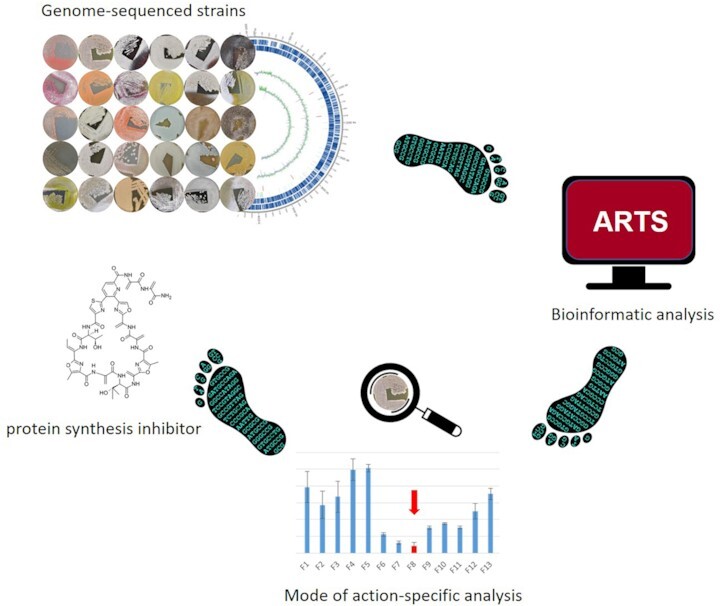
Ψ-footprinting approach.

## INTRODUCTION

According to the World Health Organization (WHO), multidrug resistance is one of the major threats to public health (1). While the number of infections caused by multidrug-resistant bacteria is constantly increasing, there has been a steady decline in the discovery and development of new druggable anti-infectives over the past years (2,3). Thus, there is an urgent need for novel antibiotic active substances. One of the major challenges in current antibiotic screening programs is the fast dereplication of already known compounds. Indeed, most antibiotics were discovered during the golden age of antibiotic discovery from the 1940s to the 1960s (4). Back then, mainly the classical screening approach was applied, which included the isolation of soil-derived microorganisms that were screened for antimicrobial activity, a procedure known as the Waksman platform. However, many years of repeated application of this screening procedure mainly led to the rediscovery of already known substances, making it necessary to develop and apply new strategies for drug discovery. One such new approach, which we also refer to in the current manuscript, is represented by the mode of action (MoA)-based *Bacillus subtilis* bioreporter panel, recently reported by Wex *et al.* (5), which allows for fast and efficient combined bioactivity and MoA screening of antibiotic producer strains. The bioreporter assay employs selected biomarkers indicative of interference with the main bacterial metabolic pathways and thereby enables to selectively follow up and prioritize metabolic activities of interest (5). This MoA-based bioreporter assay was used to screen strains from the Tübingen actinomycetes strain collection for novel natural compounds (5). Preceding this study, the Tübingen strain collection had been subjected to a bioactivity test using the newly developed 96-well microplate system, designed to rapidly screen actinomycetes strain collections in agar plug assays (6). From a set of 500 natural product producers, 270 strains showed antibiotic activity against *B. subtilis* and were tested against the bioreporter panel sensing different types of metabolic stresses. Thereby, the bioreporter *B. subtilis* P*_bmrC_-lacZ* selectively responded to ribosomal stalling as a metabolic stress signal (5). Overall, six strains from the Tübingen strain collection, namely Tü 2975, Tü 3180, Tü 6430, KNN 49.3e, Tü 2108, and A 4/2 induced the *B. subtilis* P*_bmrC_-lacZ* bioreporter and were shown to produce protein synthesis inhibitors (PSIs), whereby Tü 2975 produced pristinamycin, Tü 3180 griseoviridin/viridogrisein, Tü 6430 pactamycin, KNN 49.3e amicetin, Tü 2108 berninamycin C, and A 4/2 cycloheximide (5). Cycloheximide was probably found rather accidentally, as A 4/2 only caused an ambiguous signal in the bioreporter assay and cycloheximide is known to act as eukaryotic PSI exclusively. In general, the bioreporter panel proved to be a highly efficient and sensitive tool for rapid compound identification and target deconvolution and allowed to identify natural products from strains of the Tübingen strains collection that had not been assigned to them before.

Other discovery efforts focus e.g. on data mining strategies involving genetic and analytical tools to exploit the so far hidden biosynthetic potential of the microbial natural compound producers. The most recent advances and achievements with a special focus on genome-guided discovery efforts have just recently been reviewed by Scherlach and Hertweck (7).

Regarding the antibiotic production capacity, actinomycetes are recognized as the most important source since they are the origin of up to 70% of today's therapeutically applied antibiotic agents (8–10). Even if these microorganisms have been extensively exploited over the past decades, they are still valuable sources for novel natural compounds. Bioinformatic analysis of >170 000 bacterial genomes and several thousand metagenome sequences suggests that overall only 3% of the genomic potential for natural product biosynthesis has been discovered so far and that especially the genus *Streptomyces*, belonging to the heterogeneous group of the actinomycetes, has a huge untapped potential for the production of novel secondary metabolites (11). The antibiotic encoding genes are usually organized as biosynthetic gene clusters (BGCs) in the genome of the producer strains (12). On average, *Streptomyces* genomes harbor 40 BGC (13), whereby the majority (∼90%) of all clusters is suggested to be silent, implying that the respective antibiotics are not produced under standard lab conditions (14). Bioinformatic tools and databases, such as antiSMASH, enable the prediction of BGCs in genome sequences (15) and allow genome mining of natural compound producers. Producer strains can be prioritized bioinformatically, e.g. by screening genome sequences for potential self-resistance genes. Specialized tools and databases such as the ‘Antibiotic Resistant Target Seeker’ (ARTS) allow for an automated prediction for potential resistance genes (16). The scientific background behind this data-mining attempt is that self-resistance mechanisms of natural compound producers can provide information of the MoA of the produced bioactive compound and can help to identify the encoding BGCs. For example, the molecular target of the cystobactamids was identified by studying the self-resistance mechanism in the myxobacterial producer organism *Cystobacter* sp. (17). Here, the gene *cysO* encodes a putative protein belonging to a family of pentapeptide repeat protein with a proven resistance function against type II topoisomerase drugs. *cysO* is part of the cystobactamid BGC and the implied function as potential resistance factor against type II topoisomerase drugs paved the way to disclose the MoA of the new family of cystobactamid antibiotics as topoisomerase inhibitors (18). The cystobactamid example shows that the search for potential resistance genes can help predict the MoA of the associated antibiotic and identify its encoding BGC. A similar example is shown by the anticancer agent salinosporamide A from *Salinispora tropica* CNB-440, a compound identified as a proteasome inhibitor due to the presence of a redundant proteasome subunit gene as part of the salinosporamide BGC (19). Following up on this observation, 86 *Salinispora* genomes were browsed for the occurrence of duplicated housekeeping genes that co-localized with BGCs. Thereby, a thiotetronic acid antibiotic BGC was found to be co-localized with a putative fatty acid synthase (FAS) resistance gene, which disclosed the MoA of the thiotetronic acid compounds as FAS inhibitors (20). Accordingly, for the antibiotics novobiocin (gyrase B), platensin (FabB/F) and griselimycin (DnaN), it is known that target-duplicated resistance genes are co-clustered with their encoding BGCs. These examples demonstrate the rationale of target-directed genome mining strategies (20). ARTS is a computational tool that takes this observation into account and enables the computer-aided search for potential self-resistance genes in bacterial genome sequences (21). ARTS predicts putative resistance genes based on three criteria: (i) duplication of genes, (ii) localization within a BGC and (iii) evidence for a horizontal gene transfer event (HGT). A defined set of core (‘housekeeping’) genes is used as a reference to identify essential core genes, defines duplication thresholds and infers HGT events. Traceability of HGT was implemented as a search criterion since antibiotic resistance genes are often transferred via HGT amongst bacteria and transfer events can be deduced from differences in the codon usage or GC content of DNA sequences compared to the rest of the genome (16). Altogether, ARTS represents an innovative tool that allows for a target-directed genome mining approach.

Based on the above-mentioned knowledge on BGC identification, we aimed to establish a genome sequence-based strategy to prioritize natural compound producers with a special emphasis on producers of PSIs. We focused on PSIs since the bacterial ribosome is a hotspot for many successful antibiotics, involving important drugs from different compound classes, e.g. aminoglycosides, macrolides, tetracyclines, lincosamides, pleuromutilins thiopeptides, or streptogramins (22). The bacterial ribosome is one of the most conserved functional units of the bacterial cell. It consists of the small 30S and the large 50S subunit, whereby the subunits are made up of >50 ribosomal proteins (r-proteins) and three rRNAs (23S, 16S and 5S). Ribosomal RNA represents the main target for antibiotics (23). A prominent example of a PSI is the aminoglycoside antibiotic streptomycin, which inhibits protein synthesis by interacting with the 16S rRNA and 20 ribosomal proteins of the 30S subunit (24). Streptomycin irreversibly binds to the 16S rRNA and the S12 protein, which are the main target sites (25). It inhibits the initiation process of protein synthesis (26) and causes decoding errors, which result in incorrect amino acids being incorporated into the nascent polypeptide chain (27). An example of a 50S subunit-binding PSI is the oxazolidinone antibiotic linezolid, which inhibits protein synthesis by binding to the peptidyl transferase center (PTC) of the ribosomal 50S subunit. Linezolid binds to the A-site of the PTC, interacting with various 23S rRNA nucleotides and the ribosomal S3 protein (28). Although the bacterial ribosome is a target for numerous different antibiotics, not all promising binding sites are therapeutically exploited so far, as exemplified by the PSIs evernimicin and negamycin. Negamycin is a small pseudopeptide with potent bioactivity against Gram-negative bacteria, which binds at a position near the A-site of the 30S subunit of the bacterial ribosome next to the binding site of the well-known PSI tetracycline (29). Evernimicin is an oligosaccharide antibiotic, which interacts with a specific set of nucleotides of the 23S rRNA of the 50S subunit, which are distinct from the binding sites of other known ribosome-targeting substances (30).

To search for novel natural compounds, which bind to the ribosome as a target, we have developed an *in silico* approach that allows to mine genomes for the occurrence and accumulation of protein synthesis associated-self-resistance genes. This method is called 'psi' protein synthesis inhibitor target gene footprinting = Ψ-footprinting. The method was validated by analyzing numerous genome sequences of known and recently identified PSI producers and then challenged with over 400 actinomycetes genomes from the DSMZ strain collection. From these, 15 strains were selected and probed for the production of natural compounds by HPLC-MS and protein synthesis inhibiting bioactivities by *in vitro* transcription/translation assays. For 12 of the 15 strains, protein synthesis inhibiting bioactivity was detected and for four of them the PSI was identified, proving the predictive power of the Ψ-footprinting approach.

## MATERIALS AND METHODS

### Bacterial strains, plasmids and cultivation conditions

The bacterial strains and plasmids used in this study are listed in Table 1. *Escherichia coli* NovaBlue (Novagen) was used as a cloning strain for the plasmid pET28-eGFP and *E. coli* BL21(DE3)pLysS (Novagen) was used for protein overexpression. For *in vitro* transcription/translation assays, *E. coli* strains were cultivated as reported previously (31). For cultivation and isolation of genomic DNA, actinomycetes strains were cultivated in 50 ml of R5 medium (32) in 500-ml Erlenmeyer flasks (with steel springs) on an orbital shaker (180 rpm) at 28°C.

**Table 1.** Bacterial strains and plasmids

Bacterial strain	Description	Source or reference
***Escherichia coli***		
*E. coli* NovaBlue	*endA1, hsdR17* (rK12^–^ m_K12_ +), *supE44, thi-1, recA1, gyrA96, relA1, lac*, F' [*pro*AB, *lacI*^q^, *lac*ZΔM15, Tn*10*]; Tet^R^	Novagen
*E. coli* BL21(DE3)/pLysS	F^–^*ompT hsdS_B_*(r_B_^–^ m_B_^–^) *gal dcm* (DE3) pLysS (Cm^R^)	Novagen
***Bacillus subtilis***		
*B. subtilis* ATCC 6051	Wild-type	American Type Culture Collection (ATCC)
*B. subtilis* 1S34	*amyE*::pHJS105-P*_bmrC_-lacZ*	(5)
**Actinomycetes strains**		
*Streptomyces coelicolor* M1146	Δ*act* Δ*red* Δ*cpk* Δ*cda*	(53)
*Streptomyces* sp. Tü 2108	Wild-type	Tübingen strain collection
*Streptomyces* sp. Tü 2975	Wild-type	Tübingen strain collection
*Streptomyces* sp. Tü 3108	Wild-type	Tübingen strain collection
*Streptomyces* sp. Tü 6430	Wild-type	Tübingen strain collection
*Streptomyces* sp. A 4/2	Wild-type	Tübingen strain collection
*Streptomyces* sp. KNN 49.3e	Wild-type	Tübingen strain collection
*Nocardioides albertanoniae* DSM 25218	Wild-type	DSMZ strain collection
*Micromonospora aurantiaca* DSM 43813	Wild-type	DSMZ strain collection
*Micromonospora purpureochromogenes* DSM 43821	Wild-type	DSMZ strain collection
*Actinomadura atramentaria* DSM 43919	Wild-type	DSMZ strain collection
*Lentzea albidocapillata* DSM 44073	Wild-type	DSMZ strain collection
*Rhodococcus koreensis* DSM 44498	Wild-type	DSMZ strain collection
*Rhodococcus pyridinivorans* DSM 44555	Wild-type	DSMZ strain collection
*Saccharopolyspora flava* DSM 44771	Wild-type	DSMZ strain collection
*Rhodococcus kroppenstedtii* DSM 44908	Wild-type	DSMZ strain collection
*Williamsia marianensis* DSM 44944	Wild-type	DSMZ strain collection
*Jiangella alkaliphila* DSM 45079	Wild-type	DSMZ strain collection
*Hoyosella altamirensis* DSM 45258	Wild-type	DSMZ strain collection
*Geodermatophilus nigrescens* DSM 45408	Wild-type	DSMZ strain collection
*Actinokineospora cianjurensis* DSM 45657	Wild-type	DSMZ strain collection
*Micromonospora violae* DSM 45888	Wild-type	DSMZ strain collection
**Plasmid**		
pET28a-eGFP	reporter plasmid (pET28a derivative) for *in vitro* transcription/translation assay; Kan^R^	(31)

For antibiotic production analyses, strains were cultivated in 50 ml inoculum medium (R5) at 28°C in 500 ml Erlenmeyer flasks (with steel springs) on an orbital shaker (180 rpm). After three days of cultivation, 5, 10 or 50 ml preculture was inoculated into 50, 100 or 200 ml of production medium (Supplementary Table S1), respectively. Cultures were grown for 4, 7 or 10 days at 28°C in 500 ml or 1 l Erlenmeyer flasks (with steel springs) on an orbital shaker (180 rpm).

### *Bacillus subtilis* bioassay

Antibiotic activity was analyzed in agar plug diffusion assays using *Bacillus subtilis* ATCC 6051 as a test organism. Agar plugs of well-grown cultures of actinomycetes strains were placed on a *B. subtilis* test plate. In total, 5 μl of apramycin (50 μg/ml; Sigma) was applied as a positive control on filter discs. Plates were incubated overnight at 37°C and antibiotic activity was determined by measuring the diameter of the inhibition zone around the agar plugs and filter discs, respectively.

### Target-specific bioreporter assay

Impairment of protein synthesis, more specifically the occurrence of translation arrest, was determined using the bioreporter strain *B. subtilis* 1S34 P*_bmrC_-lacZ* and agar-plug reporter assays were carried out as reported previously (5). Briefly, actinomycetes strains were cultivated for 7–10 days at 28°C on the indicated agar plates. Then, grown agar plugs of the well-grown strains were transferred to an empty petri dish before embedding them in *B. subtilis* 1S34 P*_bmrC_*-*lacZ*-containing soft agar. The agar was supplemented 150 μg/ml X-Gal (Thermo Scientific), that gives blue colorization in plates after promoter induction. Extracts or purified compounds were directly spotted on solidified soft agar plates containing *B. subtilis* 1S34 P*_bmrC_-lacZ*. Antibiotic discs containing 10 μg of chloramphenicol (Oxoid) were used as a positive control. Agar plates were analyzed for promoter induction after overnight incubation at 37°C. P*_bmrC_* induction was indicated by a blue halo surrounding the zone of inhibition of the respective test sample.

### Genomic DNA isolation and sequencing

Isolation of genomic DNA and genome sequencing of the strains Tü 2975, Tü 3180, Tü 6340 and KNN 49.3e was performed as described in Hennrich *et al.* (33) and for strains Tü 2108 and A 4/2 as described in Zecher *et al.* (34). Genome sequence-specific information is listed in Supplementary Table S2.

### Type Strain Genome Server (TYGS)

The phylogenomic analysis of selected strains was carried out with the Type (Strain) Genome Server (TYGS) v. 1.0, a free bioinformatics tool for whole-genome-based taxonomic analysis (35). Phylogenetic classification using TYGS is based on a genome database that contains the genomic, taxonomic, and nomenclatural data of all currently available type strains. The database is constantly updated (https://tygs.dsmz.de/). The TYGS platform allows phylogenetic analyses based on the full-length genome sequence of the strain of interest, which is compared with a database of type strain genomes. Thereby, TYGS provides information on the similarity of the strain to its nearest related type strain with the help of digital DNA:DNA hybridization (dDDH) values calculated by the Genome-to-Genome Distance Calculator (GGDC) 2.1 (http://ggdc.dsmz.de) (36) and determines differences in genomic G + C contents.

### ARTS analysis

The Antibiotic Resistance Target Seeker (ARTS) web tool v. 2.0 (21) was used to screen for potential self-resistance genes in genome sequences of natural compound producers. Genome sequences or accession numbers were submitted to the ARTS web-tool. The reference set was selected for Actinobacteria and the exploration mode was used. For all analyses, default settings were used.

### AntiSMASH analysis

The antiSMASH web tool v. 6.0 (15) was used to analyze whole genome sequences for the presence of BGCs. Gene cluster similarity is displayed in % and indicates the number of genes similar to a known cluster. Genes are similar if a BLAST alignment yields an *e*-value <1 × 10^–5^ and if sequence identity is >30%. In addition, the shortest alignment must encompass >25% of the sequence. If all genes of a known cluster can be found in the query cluster, the sequence similarity is 100%. Similarity decreases if fewer genes of the known cluster can be found in the query cluster (37). Default settings were used for all analyses.

### *In vitro* transcription/translation (*iv*TT) assay

The *in vitro* transcription/translation (*iv*TT) assay was performed as reported previously (31). Briefly, an S12 extract was prepared from *E. coli* BL21(DE3)pLysS and mixed with nucleotides and amino acids as substrates for the transcription and translation process. Fructose-1,6-bisphosphate was added as an energy supply and pET28-egfp as reporter plasmid. In total, 85 μl of 2.5× *iv*TT buffer, 60 μl of the S12 extract, 20 μl of an amino acid mixture (2 mM each of all 20 proteinogenic amino acids), and 20 μl of an energy mixture (1.2 mM AMP, 0.85 mM each of CMP, GMP, and UMP, 0.5 mM IPTG, 34 mg/ml l-5-formyl-THF and 33 mM fructose-1,6- bisphosphate) served as the master mix for one sample of the *iv*TT assay (31). To test the actinomycetes culture extracts for translation inhibition, 30 μl of methanolic extracts were added to each well of a 96-well microtiter plate and incubated for 30 min at 37°C to evaporate the methanolic solvent. The master mix and 10 μl of the reporter plasmid pET28-egfp (300 ng/μl) were added to each well to a final volume of 100 μl, thereby starting the reaction. All measurements were performed in triplicate using the same preparation of *E. coli* S12 extract. As positive controls, 5 μl of tetracycline (15 mg/ml; Sigma) or 5 μl of apramycin (50 mg/ml; Sigma) were used. The assay was incubated for approximately 2 h at 37°C. Fluorescence deriving from eGFP expression was measured at 485/520 nm excitation/emission with a Tecan Infinite M200 Pro device and analyzed with the i-control™ Microplate Reader Software 1.11 (Tecan).

### Compound extraction from actinomycetes cultures

After cultivation of actinomycetes strains for 1–10 days in production medium, 5 or 50 ml of culture were extracted with 5, or 50 ml ethyl acetate, respectively, for 30 min at room temperature. Ethyl acetate samples were evaporated *in vacuo* completely and then redissolved in 0.25 or 1.25 ml of methanol, respectively. Methanolic extracts were used for bioassays and high-performance liquid chromatography/mass spectrometry (HPLC–MS) analysis, were stored at 4°C or frozen at −20°C for later analysis. As controls, pure media extracts were prepared at a ratio of 1:1 media/ethyl acetate.

### Semi-preparative purification by HPLC

For compound isolation, semi-preparative HPLC was carried out with an Agilent Technologies 1260 Infinity (Agilent Technologies, Waldbronn, Germany). A sample volume of 100 μl was injected at a flow rate of 1.5 ml/min on a Luna C18 column (5 μm, 100 Å, 4.6 mm × 250 mm) and a linear gradient from 5% solvent A (0.1% formic acid in water) to 100% solvent B (0.1% formic acid in acetonitrile) over 25 min at 25°C. 1.5 ml volume of each fraction was collected time based every minute.

### HPLC–MS analysis

HPLC analyses were performed as described previously with a HP1090M system and ChemStation 3D software rev. A.08.03 (Agilent Technologies, Waldbronn, Germany) on a Nucleosil C18 column (5 mm, 125 mm × 3 mm) fitted with a precolumn (20 × 3 mm) and with a flow rate of 850 μl/min (38). HPLC–MS was performed with an Agilent 1200 series chromatography system (binary pump, high performance autosampler, DAD-detector) coupled to a LC/MSD Ultra Trap System XCT 6330 (Agilent Technologies, Waldbronn, Germany) (38). Samples were injected onto a Nucleosil 100 C18 column (3 μm, 100 × 2 mm) fitted with a precolumn (3 μm, 10 × 2 mm) at a flow rate of 400 μl/min and a linear gradient from 10% solvent A (0.1% formic acid in water) to 100% solvent B (0.06% formic acid in acetonitrile) over 15 min at 40°C.

For the dereplication process, the UV-DAD spectra of the extracts/fractions were analyzed first for relevant signals. For this purpose, the peaks in the HPLC chromatogram were individually analyzed by comparing the chromatogram with an in-house HPLC–UV–Vis database (39). Peaks showing a coincident UV–Vis entry with a possible PSI substance were followed up. The masses of the possible PSI compounds were compared with the Dictionary of Natural Products as an external database (40). A substance was classified as ‘clearly identified’, if the match with the internal database and the external database confirmed the UV–Vis and MS criteria.

## RESULTS AND DISCUSSION

### Genome mining of strain Tü 2108 leads to the identification of a berninamycin BGC and provides information on berninamycin derivative biosynthesis

In a previous study, we established a bioreporter assay panel, suitable for MoA profiling of antibacterial agents, which are secreted into the agar by producer strains. This procedure was used to screen strains from the Tübingen actinomycetes strain collection for novel natural compounds (5). Here, we used the bioreporter strain *B. subtilis* P*_bmrC_-lacZ* that specifically responds to protein synthesis inhibition, or more precisely to ribosomal stalling, as a metabolic stress signal (5). Antibiotic activity was indicated by a zone of growth inhibition in the bioreporter lawn, while the inhibitory effect on protein synthesis was signaled by a blue halo around the inhibition zone as a result of *brmC* promoter activation that drives β-galactosidase reporter gene expression and associated color change (5).

**Figure 1.**
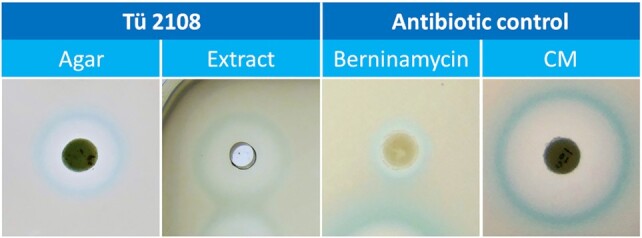
Agar-based reporter screening with samples from Tü 2108 and bioreporter test strain *B. subtilis* P_bmrC_-lacZ. 1. panel: Tü 2108-grown OM agar plug; 2. panel: 5 μl culture extract from Tü 2108 grown in R5 medium; 3.-4. panel: Pure antibiotics (50 μg berninamycin and 10 μg chloramphenicol (CM)) were prepared on filter discs as controls.

**Figure 2.**
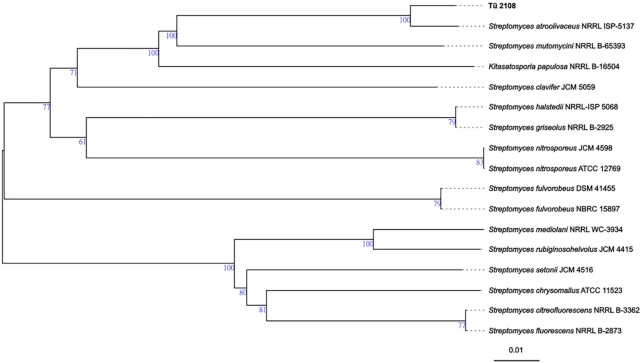
Whole-genome sequence tree generated with the TYGS web server for strain Tü 2108 and closely related species. Tree inferred with FastME from GBDP distances calculated from genome sequences. The branch lengths are scaled in terms of GBDP distance formula *d*_5_. The numbers above branches are GBDP pseudo-bootstrap support values >60% from 100 replications, with an average branch support of 84.9%.

**Figure 3.**
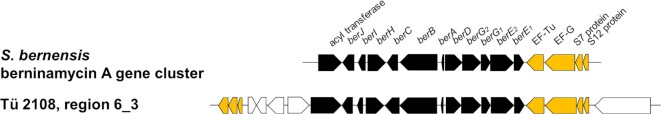
Cluster comparison between *S. bernensis* berninamycin A gene cluster and Tü 2108 region 6_3. Black: berninamycin biosynthesis genes. Yellow: ribosomal genes.

Strain Tü 2108 emerged in this MoA-informed screening process as one of the strains, which yielded a large inhibition zone and a clear blue halo (Figure 1). The implicated PSI produced by the strain was identified as berninamycin C by HPLC-MS analysis (5). For the characterization of the Tü 2108 producer organism, the genomic DNA was sequenced and the full-length genome sequence was analyzed bioinformatically for phylogenetic classification and identification of BGCs. Genome-sequence-based phylogenetic analysis was performed using the Type (Strain) Genome Server v. 1.0 (TYGS) (https://tygs.dsmz.de) (35). TYGS analysis with the genome sequence of Tü 2108 revealed that the strain is most closely related to the type strain *Streptomyces atroolivaceus* NRRL ISP-5137^T^ (Figure 2). The dDDH value (formula *d4*) between Tü 2108 and *S. atroolivaceus* NRRL ISP-5137^T^ was 84.6%, which is above the threshold of 70% commonly used for species delineation. Thus, Tü 2108 belongs to the same species as *S. atroolivaceus* NRRL ISP-5137^T^, which has already been described as a berninamycin producer strain (41). To investigate if the berninamycin BGC is present in Tü 2108, the genome sequence was analyzed with the bioinformatic tool antiSMASH v. 6.0 (15), which led to the identification of 38 BGCs, whereby the predicted BGC located on region 6_3 (BGC 6_3) showed ∼100% similarity to the berninamycin A BGC of the berninamycin producer strain *Streptomyces bernensis* (Supplementary Figure S1, Figure 3). Manual sequence analysis revealed that the antiSMASH database contained an incomplete berninamycin A BGC as a reference (MIBiG accession BGC0001472), lacking the gene *berH* from the berninamycin A BGC (*berA-J*) as initially described by Malcolmson *et al.* (42).This was found after BLAST analysis of each individual gene of BGC 6_3 from Tü 2108 and comparisons with the respective BGC genes from *S. bernensis* (Table 2). Thus, all genes essential for berninamycin biosynthesis are present in Tü 2108.

**Table 2.** Berninamycin-related genes and their described function. Tü 2108 region 6_3 ORFs is compared to the homologous *S. bernensis* genes. Gene identity (ID) and similarity (SM) of compared amino acid sequences are shown in %. ORF = open reading frame

Tü 2108 region 6_3 ORFs	Predicted gene product	Homologous *S. bernensis* gene	ID/SM (%)	Reference
01231	LmbE family protein	acyl transferase	100/100	AGN11661
01232	rRNA methyl transferase	*berJ*	99/100	AGN11674
01233	NocA homolog (C-terminal amide)	*berI*	99/99	AGN11673
01234	cytochrome P450	*berH*	99/99	KC894738
01235	lanthipeptide dehydrogenase	*berC*	99/100	AGN11672
01236	lanthipeptide dehydrogenase	*berB*	97/98	AGN11671
01237	berninamycin prepeptide gene	*berA*	98/100	AGN11670
01238	pyridine-forming	*berD*	93/96	AGN11669
01239	YcaO cyclodehydratase	*berG2*	99/99	AGN11668
01240	YcaO cyclodehydratase	*berG1*	99/99	AGN11667
01241	McbC dehydrogenase	*berE2*	98/99	AGN11665
01242	McbC dehydrogenase	*berE1*	99/98	AGN11666
01243	elongation factor Tu	*tufA/B*	100/100	AGN11675
01244	elongation factor G	*fusA*	99/100	AGN11662
01245	30S ribosomal protein S7	*rpsG*	100/100	AGN11664
01246	30S ribosomal protein S12	*rpsL*	99/100	AGN11663

### Bioinformatic analyses disclose that the berninamycin BGC is surrounded by potential self-resistance genes

Berninamycin belongs to the class of pyridine-containing thiopeptides and targets the 50S ribosomal subunit in a similar manner as described for thiostrepton (42). Several berninamycin producer strains are known, whereby the production of berninamycin derivatives differs in the various producer strains (Supplemental material Figure S3). A comparison between the known berninamycin BGC from *S. bernensis* and the BGC from Tü 2108 revealed that the predicted gene products show high amino acid sequence homologies (90–100%) amongst each other (Table 2). To identify potential self-resistance genes for strain Tü 2108, the genome sequence was analyzed with the bioinformatics software tool ARTS v. 2.0. As mentioned above, ARTS detects potential self-resistance genes based on the following individual three criteria: (i) duplicated core genes, (ii) core genes localized within a BGC and (iii) core genes with incongruent phylogeny (16). For Tü 2108, the suggested self-resistance genes were sorted with regard to genes associated with protein synthesis as a metabolic function. Concerning protein synthesis associated genes, ARTS employs a reference set of 100 core genes related to protein synthesis (16). For Tü 2108 it was found that 15 protein synthesis-associated genes met the above mentioned criteria for resistance patterns: In total, nine duplicated core genes, 13 core genes located in a BGC, and six phylogenetically distinct core genes were predicted (Table 3). The majority of the detected core genes present in a BGC, mapped to the berninamycin cluster BGC 6_3 (Table 2). Here, the core genes are organized at the left and right borders of the berninamycin BGC 6_3 (Figure 3) and code for potential ribosomal proteins (L4, S12, S10, uS7), as well as elongation factors G and TU (Table 2). For instance, ORF 01243 encoding the putative elongation factor TU (EF-TU) was duplicated and phylogenetically distinct from another putative EF-TU encoding gene (TIGR00485) present in the Tü 2108 genome. A similar genetic organization was found for the berninamycin A BGC (*berA-J*) in *S. bernensis* (Figure 3). Notably, also a *berJ* homologous gene is present within BGC 6_3 of Tü 2108, which codes for a potential 23S rRNA methyltransferase, an enzyme well-known for conferring resistance to ribosome-targeting drugs (43). However, this gene, which most likely represents the actual berninamycin self-resistance gene, was not detected with ARTS, which is due to the fact that it was not included in the list of core genes. The predominant occurrence of these protein synthesis-associated core genes in the proximity of the berninamycin BGC indicates a potential linkage to self-resistance mechanisms. This finding supports the hypothesis that resistance genes can provide an indication of the mode of action of a BGC-encoded secondary metabolite.

**Table 3.** Protein synthesis core genes of Tü 2108 identified with ARTS. Following criteria are listed: gene duplication, phylogenetic gene difference, occurrence of core genes in a biosynthetic gene cluster (BCG). +: criterion fulfilled, –: criterion not fulfilled. For BGC prediction, only region 6_3 (berninamycin BGC) is listed

Gene	Description	Duplication	Phylogeny	BGC	Accession ID
*rpsR*	30S ribosomal protein S18	+	+	+	TIGR00165
*yciO*	tRNA threonylcarbamoyl adenosine modification protein, Sua5/YciO/YrdC/YwlC family	+	+	+	TIGR00057
*rpmF*	50S ribosomal protein L32	+	+	+	TIGR01031
*rpmE*	50S ribosomal protein L31	+	+	+	TIGR00105
*tufA/B*	elongation factor Tu	+	+	6_3	TIGR00485
*rpmB*	50S ribosomal protein L28	+	-	+	TIGR00009
*rpmG*	50S ribosomal protein L33	+	-	+	TIGR01023
*rpsD*	50S ribosomal protein L4	-	-	6_3	TIGR03953
*rpsL*	30S ribosomal protein S12	-	-	6_3	TIGR00981
*rpsJ*	30S ribosomal protein S10	-	-	6_3	TIGR01049
*rpsO*	30S ribosomal protein S15	-	-	+	TIGR00952
*rpsG*	30S ribosomal protein uS7	-	-	6_3	TIGR01029
*fusA*	elongation factor G	-	-	6_3	TIGR00484
*rrsS*	tryptophan-tRNA ligase	+	+	-	TIGR00233
*rsgA*	ribosome small subunit-dependent GTPase A	+	-	-	TIGR00157

### Development of a genome sequenced-based screening approach for the identification of PSI producer strains

Based on the knowledge gained above, an *in silico* screening approach was developed, capitalizing on the occurrence of protein synthesis associated self-resistance genes to evaluate the genetic potential of bacterial strains to produce a PSI. To validate the approach, genome sequences of 47 known PSI producers were analyzed with ARTS. Here, it was found that several known PSI producers showed high numbers (>20) of protein synthesis core genes, fulfilling at least one of the three criteria mentioned for the prediction of self-resistance genes, defined as ‘*hit genes*’. For example, the anisomycin producer *Streptomyces roseochromogenus* displayed 42 hit genes and the erythromycin producer *Saccharopolyspora erythraea* 51 hit genes (Table 4). The majority of the identified hit genes of the PSI producers were characterized as phylogenetic incongruencies (76%) and only a minor part represented core gene duplications (46%) or gene localization within a BGC (26%) (Supplementary Table S3). Among the hit genes, seven core genes appeared regularly, which were *rpsE*, *rpsL*, *rplC*, *rplD*, *rplK*, *rplQ* and *rplV* encoding for the putative ribosomal proteins S5, S12, L3, L4, L11, L17 and L22, respectively (Table 4). For all these genes, it was shown in previous work that they play an essential role in conferring resistance against PSIs. For example, mutations in *rpsE*, *rpsL*, and *rplC* resulted in altered S5, S12 and L3 proteins that conferred resistance against spectinomycin, streptomycin, and linezolid/tiamulin, respectively, in *E. coli* (44–46). Mutations in *rplD* and *rplV* were shown to result in altered L4 and L22 proteins, respectively, conferring resistance to erythromycin in *E. coli* (47,48). Mutations in *rplK* resulted in an altered L11 protein conferring resistance to thiostrepton in *Thermus thermophilus* (49) and mutations in *rplQ* resulted in an altered L17 protein conferring resistance to erythromycin in *B. subtilis* (50). According to these observations, the seven ribosomal genes were regarded as PSI 'resistance indicator genes’ (RI genes). The RI genes were found to be mainly present in PSI producer strains with a high number of PSI hit genes, e.g. *S. roseochromogenus* and *S. erythraea* with six and five RI genes, respectively (Table 4). However, some of the RI genes were also found in PSI producers with a lower number of hit genes, such as *Streptomyces pristinaespiralis* PR11 and *Streptomyces pactum*, with only one RI gene each. Furthermore, there were also examples of known PSI producers that showed neither a high abundance of hit genes nor any RI gene (Table 4). To examine whether the accumulation of hit and RI genes is a specific phenomenon for PSI producers, we additionally analyzed numerous genome sequences of antibiotic producers, which are known not to produce PSIs as a comparison. Thereby, it was found that the non-PSI producers had generally low numbers of protein synthesis-related hit genes (<20) and none of the strains contained any of the seven RI genes (Table 5). Thus, we concluded that if a bacterial strain has (i) a large number of PSI hit genes (>20) and in addition (ii) two or more RI gene(s) it is likely to be a PSI producer strain.

**Table 4.** List of protein synthesis inhibitor (PSI) producer strains and their respective PSIs. Strains are ordered according to the occurrence of protein synthesis ‘hit genes’. Abundance of ‘resistance indicator’ (RI) genes/encoded proteins is highlighted as plus. Gene/protein absence is indicated as minus

Strain	Antibiotic (PSI)	Hit genes	*rpsE*/S5	*rpsL*/S12	*rplC*/L3	*rplD*/L4	*rplK*/L11	*rplQ*/L17	*rplV*/L22	RI genes	Reference
*Thermus thermophilus* HB8	Paromomycin	75	+	+	-	+	+	-	-	4	AP0082
*Saccharopolyspora erythraea* NRRL 2338	Erythromycin	51	+	+	-	+	+	+	-	5	NC_009142
*Micromonospora echinospora* DSM 43816	Gentamicin	49	-	+	-	-	-	+	+	3	NZ_LT60748
*Kitasatospora setae* KM-6054	Kirromycin	48	-	-	+	+	+	+	+	5	AP010968
*Sphaerisporangium cinnabarinum* ATCC 31213	Dityromycin	47	-	+	-	+	-	-	-	2	QAPD00000000
*Micromonospora inyonensis* NRRL 3292	Sisomicin	46	-	-	-	-	-	+	+	2	GCA_900091415
*Brevibacillus brevis* X23	Edeine	43	+	-	-	+	-	-	-	2	NZ_CP023474
*Streptomyces roseochromogenus*	Anisomycin	42	+	+	+	+	-	+	+	6	NZ_CM0022
*Micromonospora carbonaceae*	Evernimicin	40	+	-	-	-	-	-	+	2	NZ_CP058322
*Streptomyces cattleya* DSM 46488	Kirromycin	28	+	+	-	+	-	-	+	4	CP003219
*Streptomyces cattleya* NRRL 8057	Kirromycin	27	-	+	-	+	-	-	+	3	FQ859185
*Rhodococcus erythropolis* PR4	Chloramphenicol	24	-	-	-	+	-	-	+	2	AP008957
*Rhodococcus pyridinivorans* SB3094	Chalcomycin	21	-	+	-	+	-	-	+	3	CP006996
*Streptomyces griseochromogenes* ATCC 14511	Blasticidin S	19	-	-	-	-	-	-	-	-	NZ_CP0162
*Xenorhabdus nematophila* SII	Odilorhabdins	19	+	-	-	-	+	-	-	2	NZ_CP060401
*Streptomyces kasugaensis* AM-2504	Dityromycin	17	-	-	-	-	-	-	-	-	SIXH01000000
*Streptomyces rimosus* ATCC 10970	Oxytetracyclin	17	-	+	-	-	-	-	-	1	NZ_CP0236
*Streptomyces bikiniensis* NRRL B-1049	Chalcomycin	15	-	-	-	-	-	-	-	-	JNWL00000000
*Streptomyces collinus* Tü 365	Kirromycin	15	-	-	-	-	-	-	-	-	CP006259.1
*Streptomyces lateritius* JCM 4389	Granaticin	15	-	-	-	-	-	-	-	-	BMTO00000000
*Kitasatospora aureofaciens* DM-1	Chlortetracycline	14	-	-	-	-	-	-	-	-	NZ_CP0205
*Streptomyces albulus* NK660	Anisomycin	14	-	-	-	-	-	-	-	-	NZ_CP0075
*Streptomyces pristinaespiralis* PR11	Pristinamycin	14	-	+	-	-	-	-	-	1	CP059696
*Streptomyces scabiei* 87.22	Bottromycin A2	14	-	-	-	-	-	-	-	-	NC_013929
*Streptomyces albireticuli* MDJK11	Spiramycin	13	-	-	-	-	-	-	-	-	NZ_CP0217
*Streptomyces globosus* LZH-48	Factumycin	13	-	-	-	-	-	-	-	-	CP030862
*Streptomyces lincolnensis* NRRL 2936	Lincomycin	13	-	-	-	-	-	-	-	-	NZ_CP016438
*Streptomyces pactum* ACT12	Pactamycin	13	-	+	-	-	-	-	-	1	NZ_CP019724
*Streptomyces venezuelae* ATCC 10712	Chloramphenicol	13	-	-	-	-	-	-	-	-	NC_018750
*Streptomyces vietnamensis* GIM4.0001	Granaticin	13	-	-	-	-	-	-	-	-	NZ_CP0104
*Streptomyces viridochromogenes* Tü 57	Avilamycin	13	-	-	-	-	-	-	-	-	NZ_AMLP0000000
*Streptomyces xinghaiensis* S187	Neomycin	13	-	-	-	-	-	-	-	-	NZ_CP023202
*Streptomyces ambofaciens* DSM 40697	Midecamycin	12	-	-	-	-	-	-	-	-	NZ_CP012949
*Streptomyces chrestomyceticus* TBRC 1925	Paromomycin	12	-	-	-	-	-	-	-	-	JAEAGG010000010
*Streptomyces pristinaespiralis* ATCC 25486	Pristinamycin	12	-	-	-	-	-	-	-	-	CM000950
*Streptoverticillium mobaraenses* NBRC 13819	Pulvomycin	12	-	-	-	-	-	-	-	-	NZ_CP072827
*Streptomyces lavendulae* CCM 3239	Streptothricin	11	-	-	-	-	+	-	-	1	NZ_CP024985
*Streptomyces lincolnensis* NRRL 2936	Lincomycin	11	-	-	-	-	-	-	-	-	NZ_CP0164
*Streptomyces antibioticus* ATCC11891	Oleandomycin	10	-	-	-	-	-	-	-	-	NZ_CP050692
*Streptomyces bottropensis* ATCC 25435	Bottromycin A2	10	-	-	-	-	-	-	-	-	NZ_ARTP0000000
*Streptomyces laurentii* ATCC 31255	Thiostrepton	10	-	-	-	-	-	-	-	-	AP017424
*Streptomyces vinaceus* NRRL ISP-5257	Viomycin	10	-	-	-	-	-	-	-	-	NRRL ISP-5257
*Streptomyces violaceoruber* S21	Viomycin	10	-	-	-	-	-	-	-	-	NZ_CP020570
*Streptomyces fradiae* ATCC 10745	Neomycin	9	-	-	-	-	-	-	-	-	NZ_CP023696
*Streptomyces griseus* NBRC 13350	Streptomycin	8	-	-	-	-	-	-	-	-	NC_010572
*Streptomyces sparsogenes* ATCC 25498	Sparsomycin	8	-	-	-	-	-	-	-	-	MAXF00000000

**Table 5.** List of non-PSI producer strains and their known antibiotic products. Strains are ordered according to the occurrence of protein synthesis ‘hit genes’. Abundance of ‘resistance indicator’ genes/encoded proteins is highlighted as plus. Gene/protein absence is indicated black minus

Strain	Antibiotic (non-PSI)	Hit genes	rpsE/S5	*rpsL*/S12	*rplC*/L3	*rplD*/L4	*rplK*/L11	*rplQ*/L17	*rplV*/L22	RI genes	Reference
*Actinoplanes friuliensis* DSM 7358	Friulimicin	17	-	-	-	-	-	-	-	-	CP006272
*Amycolatopsis mediterranei* S699	Rifamycin	17	-	-	-	-	-	-	-	-	CP002896
*Mycobacterium avium* 104	Glycopeptidolipid, Mycobactin	15	-	-	-	-	-	-	-	-	CP000479
*Rhodococcus jostii* RHA1	Rhodochelin	15	-	-	-	-	-	-	-	-	CP000431
*Amycolatopsis orientalis* HCCB 10007	Vancomycin	14	-	-	-	-	-	-	-	-	CP003410
*Streptomyces brunneus* CR22	Feglimycin	14	-	-	-	-	-	-	-	-	NZ_CP034463
*Streptomyces violaceusniger* Tü 4113	Nigericin	14	-	-	-	-	-	-	-	-	CP002994
*Streptomyces chartreusis* NRRL 3882	Tunicamycin	13	-	-	-	-	-	-	-	-	NZ_LT963352
*Streptomyces parvulus* 2297	Actinomycin D Dactinomycin	13	-	-	-	-	-	-	-	-	NZ_CP015866
*Streptomyces viridochrom*. DSM 40736	Phosphinothricin	13	-	-	-	-	-	-	-	-	GG657757
*Streptomyces davawensis* JCM 4913	Roseoflavin	12	-	-	-	-	-	-	-	-	HE971709
*Streptomyces formicae* KY5	Formicamycins A-M	12	-	-	-	-	-	-	-	-	NZ_CP022685
*Streptomyces avermitilis* MA-4680	Avermectin Oligomycin	11	-	-	-	-	-	-	-	-	NC_003155
*Streptomyces bingchenggensis* BCW-1	Meridamycin	11	-	-	-	-	-	-	-	-	NC_016582
*Streptomyces coelicolor* A3(2)	Actinorhodin Undecylprodigiosin	11	-	-	-	-	-	-	-	-	NC_003888
*Streptomyces lividans* TK24	Actinorhodin Undecylprodigiosin	11	-	-	-	-	-	-	-	-	NZ_GG657756
*Streptomyces malaysiensis* DSM 4137	Azalomycin F	11	-	-	-	-	-	-	-	-	NZ_CP023992
*Streptomyces niveus* NCIMB 11891	Novobiocin	11	-	-	-	-	-	-	-	-	NZ_CM002280
*Salinispora tropica* CNB-440	Salinisporamide A	11	-	-	-	-	-	-	-	-	CP000667
*Streptomyces albus* DSM 41398	Salinomycin	10	-	-	-	-	-	-	-	-	NZ_CP010519
*Streptomyces flavogriseus* ATCC 33331	Carbapenem	10	-	-	-	-	-	-	-	-	CP002475
*Streptomyces pratensis* ATCC 33331	Carbapenem	10	-	-	-	-	-	-	-	-	NC_016114
*Thermobifida fusca* YX	Fuscachelin	10	-	-	-	-	-	-	-	-	CP000088
*Streptomyces clavuligerus* ATCC 27064	Tunicamycin	9	-	-	-	-	-	-	-	-	NZ_CP027858
*Streptomyces tsukubaensis* NRRL 18488	Bafilomycin	8	-	-	-	-	-	-	-	-	AJSZ01000001
*Streptomyces fungicidicus* TXX3120	Enduracidin	7	-	-	-	-	-	-	-	-	NZ_CP023407
*Streptomyces ghanaensi*s ATCC 14762	Bafilomycin	7	-	-	-	-	-	-	-	-	DS999641
*Streptomyces pluripotens* MUSC 135	Antimicin	7	-	-	-	-	-	-	-	-	NZ_CP021080
*Frankia casuarinae* CcI3	Frankiamicin	5	-	-	-	-	-	-	-	-	NC_007777

**Figure 4.**
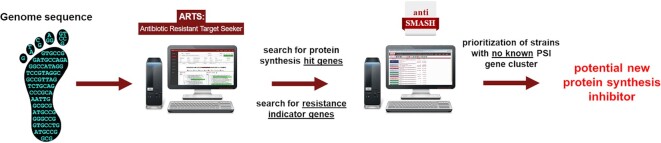
Graphic illustration of the genome sequenced-based screening approach for the identification of PSI producer strains, designated Ψ-footprinting.

Based on these findings, we devised a genome sequence-based *in silico* screening strategy for the identification of potential PSI producers, which was designated as protein synthesis inhibitor (‘psi’) target gene footprinting = Ψ-footprinting approach. The strategy involves the process of screening bacterial genome sequences with ARTS for the presence of a high number (≥20) of PSI hit genes and the abundance of RI genes (at least two), which allows for the identification of potential PSI producer strains. For dereplication purposes, the genome sequence of the identified strain is additionally analyzed with antiSMASH to sort out strains, which harbor BGCs encoding already known PSIs, allowing for the prioritization of strains likely to produce novel PSIs (Figure 4).

### Verification of the Ψ-footprinting method by analyzing PSI producer strains of the Tübingen strain collection

To apply the Ψ-footprinting method to the previously discovered PSI producers of the Tübingen strain collection (5), the genomic DNA of the strains Tü 2108, Tü 2975, Tü 3180, Tü 6430, KNN 49.3e and A 4/2 was sequenced and analyzed as described above. A 4/2 was not further evaluated as it produced the eukaryotic PSI cycloheximide. Among the five strains, three (KNN 49.3e, Tü 3180 and Tü 6430) showed large numbers of hit genes, with abundances of 88, 50 and 45, respectively (Table 6). All strains harbored at least one RI gene, whereby for KNN 49.3e all seven RI genes were detected (Table 6). This showcase demonstrates that the Ψ-footprinting method enabled a genome sequence-based identification of three out of five PSI producer strains from the Tübingen strain collection.

**Table 6.** List of protein synthesis inhibitor (PSI) producer strains from the Tübingen strain collection and their respective PSIs. Strains are ordered according to the occurrence of protein synthesis ‘hit genes’. The number of ‘resistance indicator’ (RI) genes is also listed

Strain	Antibiotic (PSI)	Hit genes	RI genes
KNN 49.3e	Amicetin	88	7
Tü 3180	Griseoviridin/Viridogrisein	50	3
Tü 6430	Pactamycin	45	3
Tü 2975	Pristinamycin	16	1
Tü 2108	Berninamycin	15	2

### Ψ-footprinting method allows to prioritize potential PSI producers from the DSMZ strain collection

To apply the Ψ-footprinting approach to so far uncharacterized strains, the method was challenged with numerous genome-sequenced strains from the DSMZ strain collection. Here, the focus was on the analysis of genome sequences from rare actinomycetes in order to increase the probability of finding new substances. 406 actinomycetal genome sequences were examined. 118 genomes (30%) showed a high number of PSI hit genes (>20) (data not shown) and 141 (35%) strains had at least one RI gene (Supplementary Table S4). 110 (27%) of the strains met the two criteria for possible PSI production (≥20 PSI hit genes and at least two RI genes) and thus were candidate strains for PSI production. Genome sequences, which met these criteria, were analyzed with antiSMASH to exclude strains containing an already known PSI BGC. In total, 15 strains were prioritized for further analysis, which harbored up to 71 hit genes and five RI genes, as for example strain *Jiangella alkaliphila* DSM 45079 (Table 7).

**Table 7.** List of possible protein synthesis inhibitor (PSI) producer strains of the DSMZ strain collection. Strains are ordered according to the occurrence of protein synthesis ‘hit genes’. Abundance of ‘resistance indicator’ (RI) genes/encoded proteins is highlighted as plus. Gene/protein absence is indicated as minus

Species	DSM number	Hit gene	*rpsE*	*rpsL*	*rplC*	*rplD*	*rplK*	*rplQ*	*rplV*	RI genes
*Jiangella alkaliphila*	45079	71	+	+	-	+	-	+	+	5
*Saccharopolyspora flava*	44771	64	+	+	-	+	+	+	-	5
*Actinokineospora cianjurensis*	45657	57	+	+	-	+	+	-	-	4
*Micromonospora aurantiaca*	43813	53	-	-	-	+	+	+	+	4
*Micromonospora violae*	45888	47	-	-	-	+	-	+	+	3
*Micromonospora purpureochromogenes*	43821	40	+	+	-	-	-	-	+	3
*Lentzea albidocapillata*	44073	40	+	+	-	-	+	+	+	5
*Nocardioides albertanoniae*	25218	39	-	+	-	-	+	+	+	4
*Actinomadura atramentaria*	43919	35	-	+	-	+	-	+	+	4
*Geodermatophilus nigrescens*	45408	34	-	+	-	-	+	-	+	3
*Hoyosella altamirensis*	45258	25	+	+	-	+	-	-	+	4
*Rhodococcus pyridinivorans*	44555	23	+	-	-	+	-	-	+	3
*Rhodococcus koreensis*	44498	22	-	-	-	+	-	-	+	2
*Williamsia marianensis*	44944	22	+	-	-	+	-	-	+	3
*Rhodococcus kroppenstedtii*	44908	20	-	-	-	+	-	-	+	2

**Figure 5.**
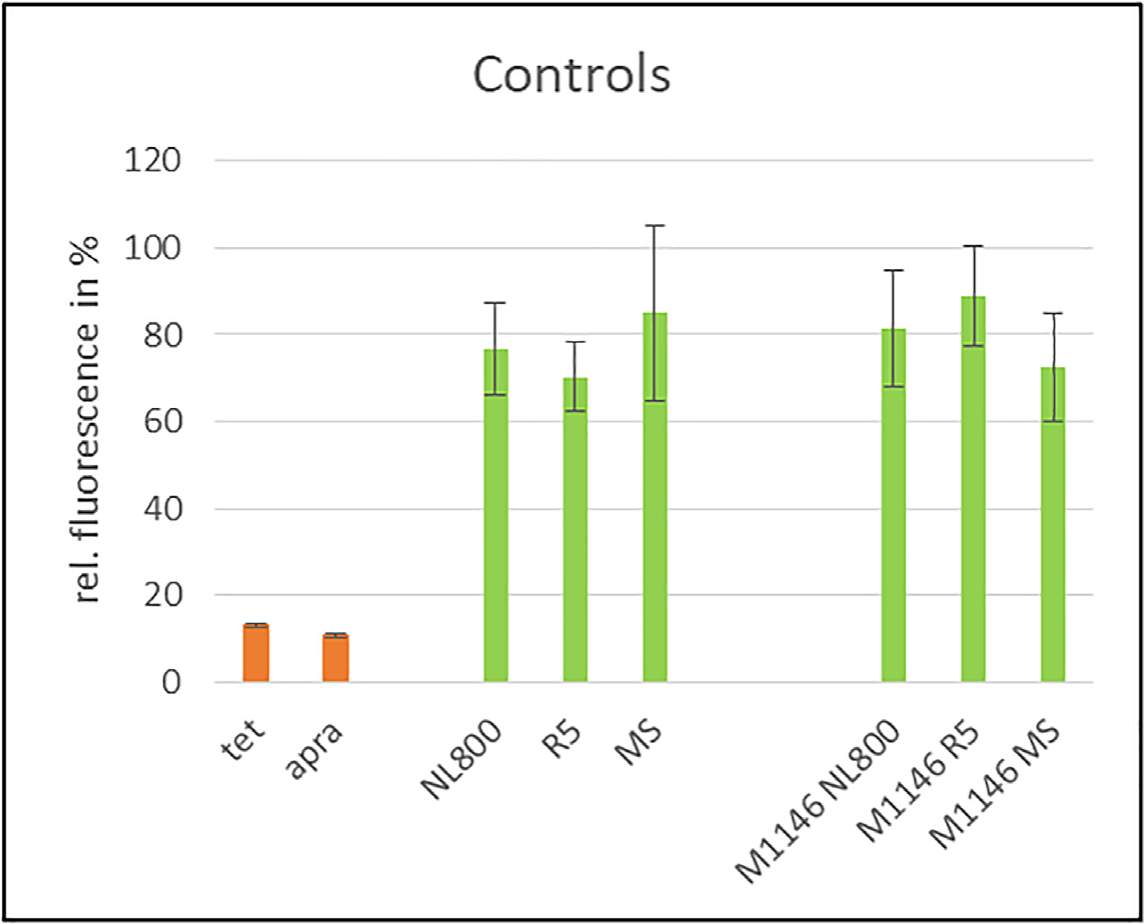
*In vitro* transcription/translation assay performed with culture extracts of controls. PSI antibiotics, tet_15_ and apra_50_ = positive control (orange), medium extracts, extracts of M1146 = negative control (green). For M1146, results are shown exemplary for samples from day 7. Measurements have been performed in triplicate using the same preparation of S12 extract.

To investigate whether the selected 15 strains produce PSIs, the strains were cultivated in three different cultivation media (NL800, MS and R5) shown to be suitable production media in previous studies (5). Culture samples were harvested at different time points (4, 7 and 10 days), extracted with ethyl acetate in a ratio of 1:1, concentrated 20-fold *in vacuo* and then redissolved in methanol. To investigate if the extracts contained substances with protein synthesis inhibiting activity, samples were analyzed using a coupled *in vitro* transcription/translation (*iv*TT) assay (31). This cell-free assay uses the *E. coli* translational machinery to express a green fluorescent protein (GFP) from a plasmid (pET28a-eGFP), reporting transcription/translation activity by fluorescence emission analysis. First, the assay was validated for the analysis of culture extracts and it was found to tolerate 30 μl of the concentrated culture extracts in a total assay volume of 100 μl without significant interference. Changes in GFP expression were monitored by measuring the fluorescence at 485/520 nm excitation/emission with a Tecan Infinite M200 Pro device and analyzing the data with the i-control™ Microplate Reader Software 1.11. To determine the maximum fluorescence value (GFP fluorescence = 100%), the assay was performed without the addition of antibiotics. As positive controls for effective protein synthesis inhibition, pure tetracycline and apramycin were used, respectively. As negative control, media extracts of the respective culture media (NL800, MS and R5) were tested to exclude that the observed inhibitory effects were caused by media components. Furthermore, extract samples from the strain *S. coelicolor* M1146 (M1146) were analyzed as a negative control. M1146 is a genetically engineered strain with deleted BGCs and thus lacking any antibiotic production (51). M1146 extracts were analyzed to exclude that the observed inhibitory effects were caused by components from the cell metabolism of actinomycetes. *Iv*TT conducted with the media extract samples as negative controls yielded 70% - 85% maximal GFP fluorescence values and thus had only a minor inhibiting effect on the assay (Figure 5). The same was observed for the culture extracts of M1146, for which the fluorescence values varied between 70% and 90% (Figure 5). The addition of 5 μl tetracycline (15 mg/ml) (tet_15_) and 5 μl of apramycin (50 mg/ml) (apra_50_) resulted in very low GFP fluorescence values of 13% and 12%, respectively, indicating a strong inhibition of the *iv*TT assay (Figure 5). Based on these control measurements, an internal threshold was set, according to which fluorescence values between 0% and 40% were considered to represent specific inhibition of the *iv*TT assay and values of 0–20% indicate strong inhibition of the assay.

**Figure 6.**
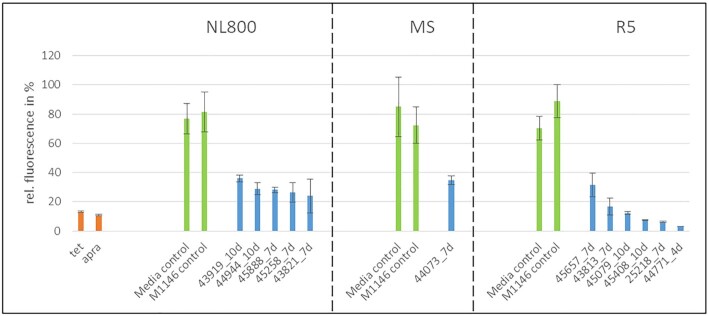
*In vitro* transcription/translation assay performed with culture extracts in NL800, MS and R5 media from 4, 7 and 10 days (optimal production time of each strain). PSI antibiotics, tet_15_ and apra_50_ = positive control (orange), medium extracts, extracts of M1146 = negative control (green), and extracts of DSM cultures (blue). Displayed are the extracts of each DSM strain that resulted in the most decrease of GFP production of the *iv*TT assay. Measurements have been performed in triplicate using the same preparation of S12 extract.

*Iv*TT assays with extract samples of strains DSM 44555, DSM 44498 and DSM 44908 showed no significant inhibition of GFP expression and thus the samples obviously did not contain protein synthesis inhibiting substances or at least they were not produced by the strains under the applied cultivation conditions (Supplementary Figure S4). *Iv*TT assays with extract samples from the other twelve strains (DSM 44944, DSM 45888, DSM 45258, DSM 43821, DSM 44073, DSM 45079, DSM 45657, DSM 43813, DSM 43919, DSM 45408, DSM 25218 and DSM 44771) yielded significantly low fluorescence values ranging from 6% - 40% relative fluorescence (Supplementary Figure S5) and thus specific inhibition of the assay. This was observed for samples obtained from all tested media, whereby data are shown for the respective best production medium (Figure 6), while the complete dataset can be found in the supplemental material (Supplementary Figure S5). *iv*TT assays were additionally carried out with eleven culture extract sample from random strains of the Tübingen strain collection, of which none led to a specific inhibition (<40% fluorescence) of the assay, demonstrating that none of the strains produced a substance with PSI activity (Supplementary Figure S6).

*Iv*TT assays with culture extracts of DSM 43813, DSM 45079, DSM 45408, DSM 25218 and DSM 44771 grown in R5 resulted in the lowest fluorescence values (<20% maximal fluorescence), which indicated strong inhibition of the GFP expression, representatively shown for sample DSM 43813 (Figure 6, Supplementary Figure S5). Samples of strains DSM 43813 and DSM 45079 from sampling time points at days 7 and 10, respectively, reduced fluorescence to values of 17% and 12%, respectively (Figure 6). Samples of strains DSM 45408, DSM 25218 and DSM 44771 showed the strongest inhibiting effect for sampling time points at days 4, 7 and 10 with residual fluorescence values of 3%, 6% and 7%, respectively (Figure 6, Supplementary Figure S5). Since culture extracts of these five strains specifically inhibited the *iv*TT assay, it could be concluded that they interfere with protein expression and may contain a PSI. Thus, using the Ψ-footprinting-based prioritization of actinomycetes strains, potential PSI production was detected by *iv*TT assays for twelve out of the 15 (80%) selected DSM strains.

### Identification of PSI substances from Ψ-footprinting-prioritized strains of the DSMZ strain collection

**Figure 7.**
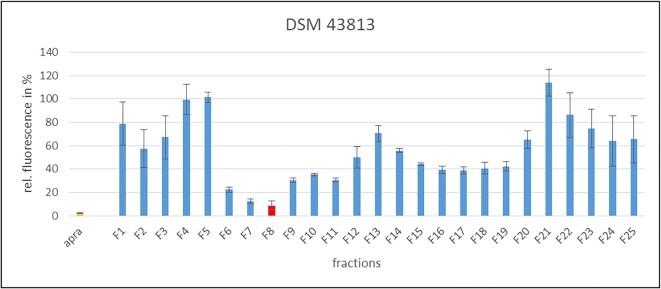
*In vitro* transcription/translation assay performed with R5 culture extract of DSM 43813 from day 7. Displayed are the fractions generated by semi-preparative HPLC (blue). PSI antibiotic apra_50_ = positive control (orange). Fraction having the greatest inhibition is shown in red. Measurements have been performed in triplicate using the same preparation of S12 extract.

**Figure 8.**
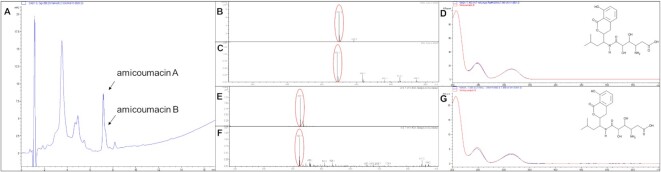
(**A**) HPLC chromatogram from fraction F8 of the R5 extract from DSM 25218. Wavelength monitoring was performed at 260 nm. Arrows indicate amicoumacin A and B specific peaks at RT 7.2 min and 7.4 min, respectively. (B, C) Mass spectra from fraction F8 of the R5 extract of the amicoumacin producer strains. Amicoumacin A peaks in positive mode (**B**) *m/z* = 424.2 [M + H]^+^ and negative mode (**C**) *m/z* = 422.1 [M-H]^–^ at RT 7.2 min are marked with red ellipses. (**D**) UV–Vis spectrum of DSM 25218 extract sample of the main peak at RT 7.2 min with amicoumacin B as reference and structure. Here, amicoumacin B was used as a reference since it was the only amicoumacin compound available in the internal database. (E, F) Mass spectra from fraction F8 of the R5 extract of the amicoumacin producer strains. Amicoumacin B peaks in positive mode (**E**) *m/z* = 425.1 [M + H]^+^ and negative mode (**F**) *m/z* = 423.1 [M-H]^–^ at RT 7.4 min are marked with red ellipses. (**G**) UV–Vis spectrum of DSM 25218 extract sample of the shoulder peak at RT 7.4 min with amicoumacin B as reference and structure. The background was subtracted due to the overexposed masses of amicoumacin A for B/C and E/F (from main peak).

In order to identify the PSIs produced by the selected and analyzed DSM strains, the culture extracts were purified by semi-preparative HPLC followed by HPLC-MS analysis of the pre-purified substances. For these analyses, we focused on samples that showed the strongest inhibition of the *iv*TT assays, which included DSM 43813, DSM 45079, DSM 45408, DSM 25218 and DSM 44771. Methanolic culture extracts were prepared from samples harvested at the optimal production time point as reported above and protein synthesis inhibiting activity was verified in *iv*TT assays. For compound purification, semi-preparative HPLC was performed with the culture extracts. The fractions were collected in time-based mode every minute for 25 min and were analyzed for PSI activity via *iv*TT. Assay analysis revealed that individual purified fractions from all five DSM strain samples (DSM 43813, DSM 45079, DSM 45408, DSM 25218 and DSM 44771) showed significant inhibition of the *iv*TT assay (Figure 7, Supplementary Figures S7-S10). Interestingly, for all five extracts, fraction 8 (F8) showed the strongest inhibition, with fluorescence values of 9%, 30%, 4%, 33% and 7%, respectively (Figure 7, Supplementary Figures S7-S10), which suggested that F8 contained a protein synthesis inhibiting substance. HPLC-MS analysis of all extract samples and F8 fractions was performed to identify the respective PSI. The HPLC chromatograms of the extract samples of F8 of DSM 43813, DSM 45408, DSM 25218, and DSM 44771 of F8 showed a prominent peak at a retention time (RT) 7.2 min, as representatively shown for the sample DSM 25218 (Figure 8A). HPLC-MS analysis of the F8 samples of DSM 43813, DSM 45408, DSM 25218 and DSM 44771 revealed one distinct peak in positive and negative mode at RT 7.2 min (Figure 8B and C). This peak showed a characteristic UV–Vis spectrum with three λ_max_ at 206 , 248 and 314 nm, respectively (Figure 8D). The peak was detected in positive mode at *m/z* = 424.2 [M + H]^+^ and in negative mode at *m/z* = 422.1 [M–H]^–^ (Figure 8B and C). The mass of *m/z* = 423 is consistent with the mass of amicoumacin A (molecular weight 423.5; molecular formula C_20_H_29_N_3_O_7_) (52). The peak at RT 7.4 shows a prominent shoulder (Figure 8A). This often results due to the presence of two similar substances, which were not separated chromatographically. The shoulder showed a UV–Vis spectrum similar to that of amicoumacin A (Fig. 8G versus D). The UV–Vis spectrum of the peak was compared with an internal database, matching the entry of amicoumacin B (Figure 8G). This peak was detected in positive mode at *m/z* = 425.1 [M + H]^+^ and in negative mode at *m/z* = 423.1 [M–H]^–^ (Figure 8E and F). The mass of *m/z* = 424 is consistent with the mass of amicoumacin B (molecular weight 424.4; molecular formula C_20_H_28_N_2_O_8_) (52). Amicoumacin A and B production have further been confirmed by high-resolution MS/MS (HRMS) analysis with F8 sample of DSM 45408, representatively (Supplementary Figure S11). The data obtained in this study (UV–Vis spectrum in combination with RT and mass of the peaks) showed that F8 of the samples DSM 43813, DSM 45408, DSM 25218 and DSM 44771 contained the PSIs amicoumacin A and B. Regarding F8 of strain DSM 45079, the mass of amicoumacin was not detected in this sample, and no mass or UV–Vis spectra of known PSIs could be assigned. It cannot be ruled out that amicoumacin was present in this sample in a very low concentration, which would fit to the lower inhibitory effect in the *iv*TT assay. On the other hand, this result could also indicate that the strain produces a novel PSI, but further cultivation and purification steps are necessary to allow accurate conclusions.

Amicoumacins are known PSIs, belonging to a class of isocoumarin antibiotics with antibacterial, antifungal, anticancer, and anti-inflammatory activities (53). They also show potent antibacterial activities against clinically relevant bacterial pathogens, such as *Helicobacter pylori* and methicillin-resistant *Staphylococcus aureus* (54,55). Amicoumacin affects bacterial growth by binding to the 30S subunit of the bacterial ribosome, specifically at the E-site codon of the mRNA, and thereby inhibits the translation process. This position is a rather unusual target site and implies a unique way of translation inhibition for amicoumacin (56). Amicoumacin has been reported as a natural product from different microbial producers, involving Gram-positive bacteria, such as certain representatives of *Bacillus* and *Nocardia* (54,57,58) but also the Gram-negative producer *Xenorhabdus bovienii* (53). In addition, our data revealed that amicoumacin is produced by various types of actinomycetes, namely *Saccharopolyspora flava* DSM 44771, *Micromonospora aurantiaca* DSM 43813, *Nocardioides albertanoniae* DSM 25218, and *Geodermatophilus nigrescens* DSM 45408, which belong to different orders of the phylum Actinobacteria, such as *Pseudonocardiales, Micromonosporales, Propionobacteriales*, and *Geodermatophilales*, respectively (Supplementary Figure S12). Thus, the ability to produce amicoumacin seems to be quite conserved across different groups of bacterial organisms, hinting at a so far unknown general biological function. The reason why we have not been able to dereplicate the substance based on BGC predictions is that the respective amicoumacin BGCs could not be identified by antiSMASH analysis. The amicoumacin BGC was described as nonribosomal peptide-polyketide hybrid BGC for the producer strain *Xenorhabdus bovieni* (53). However, the BGC is not part of the MIBiG repository and thus is not accessible as a reference in the antiSMASH database, which is why the BGCs were not identified by antiSMASH analysis for the amicoumacin producers of the DSMZ strain collection. Remarkably, also detailed manual sequence analyses did not lead to the identification of the amicoumacin BGCs. We therefore assume that the actinobacterial amicoumacin biosynthesis (high GC content bacteria) proceeds differently and is encoded by a strikingly different type of BGC than in the known producers of *Xenorhabdus* and *Bacillus* (low GC content bacteria). If so, this would speak for a convergent evolution of the amicoumacin BGCs in the phylogenetically distantly related producer strains. The fact that we have detected amicoumacin as PSI in our samples underscores the feasibility and reliability of the Ψ-footprinting strategy. The advantage of this method is that it is purely *in silico*-based. Conventional antibiotic screening approaches are generally much more labor-intensive since they involve screening a large number of strains, followed by extensive compound purifications and chemical analytics with the risk that the identified bioactive substance is already known. The Ψ-footprinting strategy is a fast and efficient method to specifically prioritize potential producers of PSIs based on genome-sequence information and thereby speeds up the lead discovery process. Until now, it was not possible to transfer this method to antibiotics with a different mode of action. The derived *in silico* strategy is so far only applicable for the prioritization of potential PSI producer strains, due to the number of core genes used as a reference set in the ARTS analysis. For the metabolic function of protein synthesis ∼100 core genes are defined in the reference set, which are significantly lower core gene numbers for other metabolic functions, such as DNA metabolism- or cell envelope-associated functions (16). Certainly, not all PSI producers can be detected with the Ψ-footprinting approach, as for example shown for *Streptomyces griseus*, a known producer of the PSI streptomycin (Table 4). Another limitation of the approach might be that it employs known RI genes as indicator genes, which could prevent the discovery of really novel PSIs with different MoAs. In this context, however, it would be possible to adopt a less stringent Ψ-footprinting run and exclude RI genes as a selection criterion, which would allow for a more explorative screening attempt. However, following the defined criteria for Ψ-footprinting, which are (i) a high number (≥20) of PSI hit genes and (ii) the additional abundance of RI genes, allows a reliable pre-selection of potential PSI producers. This was proven by the identification of twelve PSI producer strains from the 15 Ψ-selected strains in the current study, which inhibited protein synthesis in *iv*TT assays. In addition, the responsible PSI substances (amicoumacin A and B) were identified for four of these strains. Prioritized strains for which protein synthesis inhibiting activities were observed but no PSI identified yet are subject to ongoing work.

## DATA AVAILABILITY

Genome sequencing data can be found at the National Center for Biotechnology Information (NCBI) linked to the accession numbers CAKMXI010000001-CAKMXI010000042 (Tü 2108), JAKGSH010000001-JAKGSH010000010 (Tü 6430), CALNVW010000001-CALNVW010000144 (A 4/2) and CP091196 (KNN 49.3e).

## Supplementary Material

lqac055_Supplemental_File
