# Supplementary material for: Ψ-Footprinting approach for the identification of protein synthesis inhibitor producers
Source: NAR Genom Bioinform. 2022 Jul 15;4(3):lqac055. doi: 10.1093/nargab/lqac055 (PMC9290621; doi:10.1093/nargab/lqac055)
Supplement: lqac055_Supplemental_File [file lqac055_supplemental_file.pdf]

Supplementary Material

Ψ-footprinting approach for the identification of protein synthesis inhibitor producers

Franziska Handel[1,5], Andreas Kulik[1,2], Katharina W. Wex[2,5], Anne Berscheid[2,5], Julian S. Saur[3], Anika Winkler[4], Daniel Wibberg[4], Jörn Kalinowski[4], Heike Brötz-Oesterhelt[2,5,6], Yvonne Mast[1,5,7,8]*

[1]Department of Microbiology/Biotechnology, Interfaculty Institute of Microbiology and Infection Medicine, Faculty of Science, University of Tübingen, Auf der Morgenstelle 28, D-72076 Tübingen, Germany

[2]Department of Microbial Bioactive Compounds; Interfaculty Institute of Microbiology and Infection Medicine, University of Tübingen; Tübingen, Baden-Württemberg, 72076; Germany

[3]Biomolecular Chemistry; Institute of Organic Chemistry, University of Tübingen; Tuebingen, Baden-Württemberg, 72076; Germany

[4]Center for Biotechnology (CeBiTec), Bielefeld University, Universitätsstraße 27, 33615, Bielefeld, Germany

[5]German Center for Infection Research (DZIF), Partner Site Tübingen, Tübingen, Germany

[6]Cluster of Excellence Controlling Microbes to Fight Infection

[7]Department Bioresources for Bioeconomy and Health Research, Leibniz Institute DSMZ - German Collection of Microorganisms and Cell Cultures, Inhoffenstraße 7B, 38124 Braunschweig, Germany

[8]Technical University Braunschweig, Department of Microbiology, Rebenring 56, 38106 Braunschweig, Germany

*Correspondence:
Prof. Dr. Yvonne Mast
yvonne.mast@dsmz.de

Supplementary Table S1: Composition of the production media used for cultivation of the antibiotic producers. All quantities refer to 1 l.

Medium	Composition	Amount
NL500	Glycerol	10 g
	Starch, soluble	10 g
	Glucose	10 g
	Fish Meal	15 g
	Sea Salts	10 g
		pH 8.0
NL800	Glucose	5 g
	Glycerol	10 g
	Starch, soluble	10 g
	Oatmeal, Holo	58 g
	Yeast extract	2 g
	NaCl	1 g
	$CaCO_3$	1 g
		pH 7.2
MS (1)	Mannitol	20 g
	Oatmeal, Holo	20 g
R5 (1)	Sucrose	103 g
	Glucose	10 g
	K_2SO_4	0.25 g
	$MgCl_2$	10.12 g
	Casamino acids	0.1 g
	Yeast extract	5 g
	TES	5.73 g
	$CaCl_2$ x $2H_2O$	2.94 g
	KH_2PO_4	0.05 g
	L-proline	3 g
	Trace element solution	2 ml
		pH 7.4
Trace element solution	Fe x $6H_2O$	200 mg
	$Na_2B_4O_7$ x $10H_2O$	10 mg
	$(NH_4)_6Mo_7O_{24}$ x $4H_2O$	10 mg
	$CuCl_2$ x $2H_2O$	10 mg
	$MnCl_2$ x $4H_2O$	10 mg
	$ZnCl_3$	40 mg

Supplementary Table S2: Genome sequence data for all analyzed strains of the Tübingen strain collection.

	**Tü 2108**	**Tü 2975**	**Tü 3180**	**Tü 6430**	**A 4/2**	**KNN 49.3e**
**Sequencing method**	Illumina MiSeq	PacBio RSII	PacBio RSII	PacBio RSII	Illumina MiSeq	PacBio RSII
**De novo Assembly**						
contig(s)	189	1	2	10	144	1
draft sequence (in bp)	7,920,474	7,623,788	8,634,962	8,176,333	8,295,973	7,285,716
N_{50} contig	219,353	7,623,788	8,390,116	5,909,951	235,910	7,285,716
maximal length	657,624	7,623,788	8,390,116	5,909,951	511,072	7,285,716
average. length	64,286	7,623,788	4,317,481	817,633	91,077	7,285,716
coverage depth	129	87	95	50	105	123
average. G+C content (in %)	70.81	71.04	72.97	72.63	70.27	71.85
**Genome Annotation**						
coding sequences (CDSs)	7,064	6,950	7,470	8,019	7,398	7,005
tRNA	82	80	97	94	78	70
rRNA	9	18	18	18	9	9

Region	Type	From	To	Most similar known cluster		Similarity
Region 4.1	siderophore	132,675	144,459	desferrioxamin B	Other	80%
Region 4.2	lanthipeptide	207,067	230,198	niphimycins C-E	Polyketide	6%
Region 6.1	bacteriocin	44,627	54,830			
Region 6.2	lanthipeptide	115,833	141,053			
Region 6.3	thiopeptide , LAP	466,816	497,811	berninamycin A	RiPP	100%
Region 10.1	butyrolactone	90,791	101,717	lactonamycin	Polyketide	3%
Region 18.1	terpene	144,069	165,142			
Region 19.1	terpene	87,908	108,960	steffimycin D	Polyketide:Type II + Saccharide:Hybrid/tailoring	16%
Region 19.2	NRPS-like	396,004	437,854	glycinocin A	NRP	9%
Region 22.1	butyrolactone	10,280	21,194	rimosamide	NRP	14%
Region 23.1	lanthipeptide	1	21,233	neoabyssomicin / abyssomicin	Polyketide	9%
Region 24.1	T3PKS	61,371	102,495	tetronasin	Polyketide	11%
Region 24.2	melanin	175,596	186,069	melanin	Other	100%
Region 25.1	T2PKS , terpene	1	44,932	spore pigment	Polyketide	83%
Region 25.2	NRPS	110,514	136,384	kanamycin	Saccharide	1%
Region 29.1	NRPS	12,002	62,903	coelichelin	NRP	81%
Region 34.1	NRPS	1,773	59,016	mirubactin	NRP	64%
Region 34.2	terpene	65,138	90,630	isorenieratene	Terpene	100%
Region 35.1	bacteriocin	1,946	13,238			
Region 35.2	NRPS	224,143	253,561	α-lipomycin	NRP:Lipopeptide + Polyketide:Modular type I + Saccharide:Hybrid/tailoring	9%
Region 39.1	NRPS	17,756	79,861	daptomycin	NRP	10%
Region 39.2	melanin	149,593	159,970	grixazone	Terpene	61%
Region 39.3	oligosaccharide	243,028	274,376	auroramycin	Polyketide	40%
Region 41.1	terpene	50,766	77,357	hopene	Terpene	69%
Region 41.2	NRPS	156,102	210,128	coelibactin	NRP	100%
Region 46.1	PKS-like	62,303	103,322	rustmicin	Polyketide:Iterative type I	33%
Region 46.2	NRPS	174,553	209,944	crochelin A	NRP + Polyketide	11%
Region 48.1	T1PKS , terpene	104	25,382	mycinamicin II	Polyketide	19%
Region 48.2	bacteriocin	104,323	114,550			
Region 48.3	hglE-KS , T1PKS	122,166	162,855			
Region 50.1	ectoine	1	1,012	ectoine	Other	50%
Region 53.1	T1PKS	1	57,245	auroramycin	Polyketide	40%
Region 53.2	siderophore	91,851	106,441			
Region 64.1	terpene	7,667	28,689	cadaside A / cadaside B	NRP	9%
Region 67.1	NRPS	1	18,622	feglymycin	NRP	15%
Region 68.1	T1PKS	1	13,178	nigericin	Polyketide:Modular type I	44%
Region 71.1	NRPS	1	14,779			
Region 73.1	T1PKS	1	10,243			

Supplementary Figure S1: List of predicted secondary metabolite gene clusters for strain Tü 2108 identified by antiSMASH 6.0 analysis.

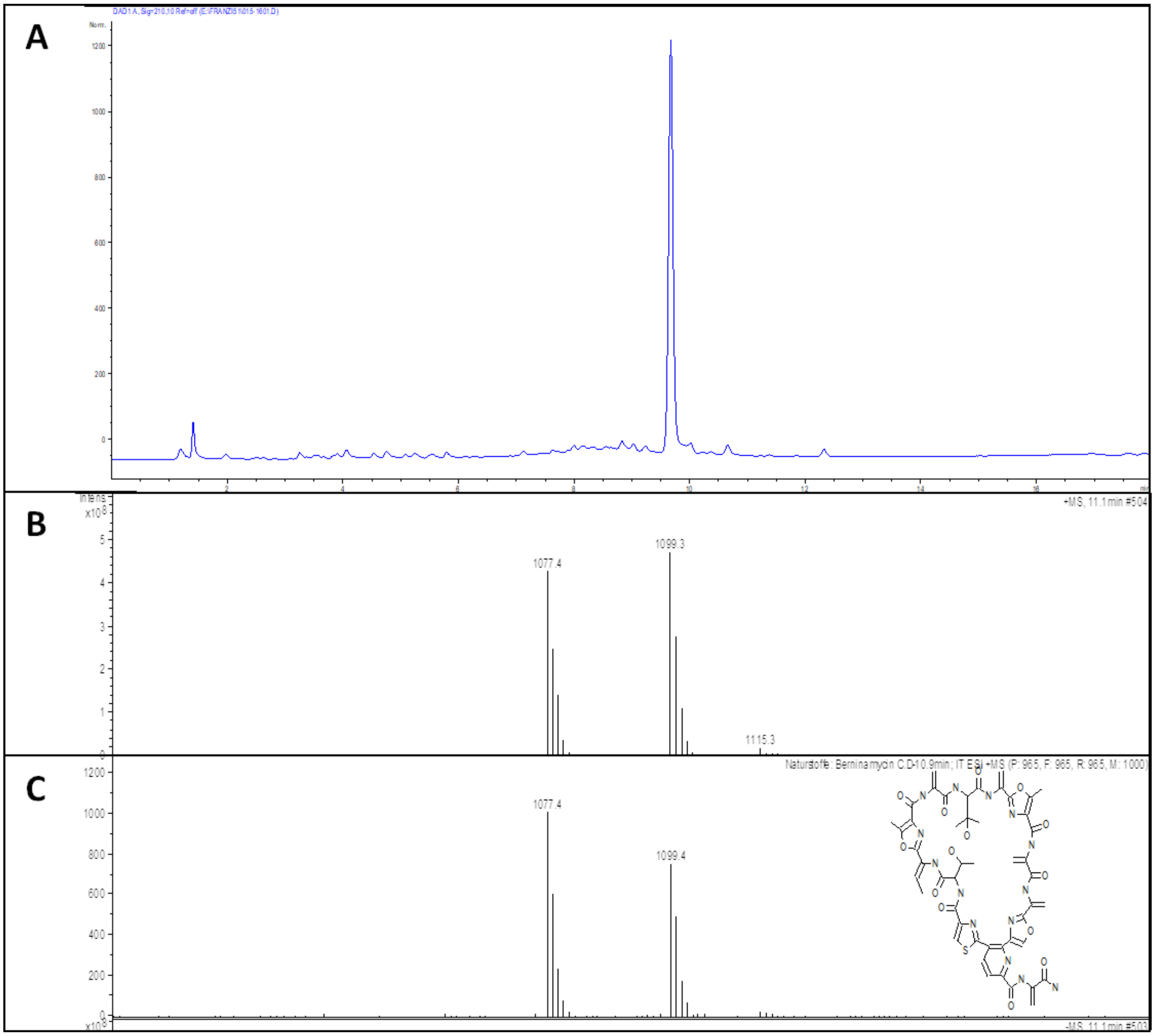

Supplementary Figure S2: (A) HPLC spectrum of the R5 culture extract from strain Tü 2108. Wavelength monitoring was performed at 210 nm. Berninamycin C specific peak at retention time (RT) 9.7 min. (B) MS data referring to peak at RT 9.7 min. Berninamycin C peaks (1077.4 [M+H]+, *m/z* 1099.3 [M+Na]+; (2)) at RT 11.1 min are shown. (C) MS reference data from berninamycin C (1077.4 [M+H]+, *m/z* 1099.4 [M+Na]+) at RT 10.9 min and corresponding chemical structure from the Dictionary of Natural Products (3).

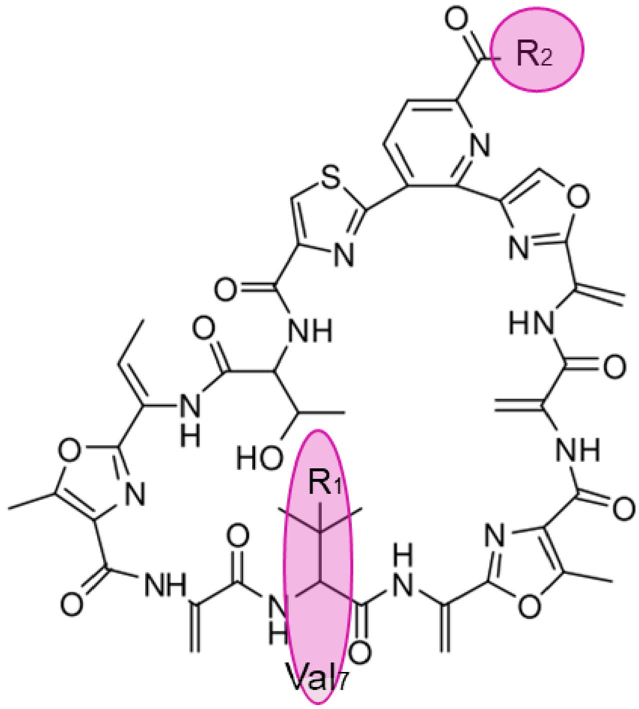

Berninamycin A: R_1 = Dha2-Dha1-NH_2, R_2 = OH
Berninamycin B: R_1 = Dha2-Dha1-NH_2, R_2 = H
Berninamycin C: R_1 = Dha2-NH_2, R_2 = OH
Berninamycin D: R_1 = NH_2, R_2 = OH
Berninamycin E: R_1 = Dha2-ala-NH_2, R_2 = OH

Supplementary Figure S3: Chemical structure of berninamycin derivatives. Berninamycin A - hydroxy group at Val7; berninamycin B - missing hydroxy group at Val7; berninamycin C - one missing dehydroalanine residue at the N-terminus, berninamycin D - two missing dehydroalanine residues at the N-terminus; berninamycin E - in place of the N-terminal dehydroalanine is an alanine residue (2, 4) Purple ellipses indicate the position of the altered berninamycin residues. Dehydroalanine (Dha), alanine (Ala), valine (Val).

Supplementary Figure S3: Additional information: Berninamycin BGC comparisons provide information on the production of compound derivatives:

Tü 2108 was found to produce berninamycin C exclusively (5) (Figure S2). *S. bernensis* produces berninamycin A-D, with berninamycin A as the major compound (6), whereas heterologous expression of the *berA-J* gene cluster from *S. bernensis* in *Streptomyces lividans* resulted in a stable berninamycin A production, while berninamycin B and D were only produced in tiny amounts and berninamycin C was not produced at all (6). *S. atroolivaceus* produces berninamycin A and E, with berninamycin A as the major compound (4, 7). Differences in berninamycin derivative production might be related to the *berA* core-prepeptide coding sequence. Malcolmson *et al.*, 2013 showed that mutations in the BerA core-prepeptide sequence result in the production of berninamycin analogs, e.g. the introduction of a T3A mutation in the *berA* gene sequence resulted in

the production of different compounds in *S. lividans,* including a substance with a C-terminal amide, an unmodified Val7 residue, and an N-terminal alanine residue (6). Thus, *berA* mutations cause structural changes in the BerA protein, which are responsible for the formation of the various different berninamycin derivatives (2, 4) (Figure S3). Since the similarity between BerA from *S. bernensis* and ORF 01237 from Tü 2108 is rather low (only 65%), the exclusive berninamycin C production of Tü 2108 is likely to be the result of the specific core prepeptide sequence encoded by ORF 01237. Thus, the results presented in this study show that Tü 2108 is a new producer strain of berninamycin and provide an explanation for the high berninamycin C production rate of Tü 2108.

Supplementary Table S3: Sum of the 790 detected hit genes from 35 analyzed genome sequences of known PSI producers and their assignment to the following criteria for self-resistance genes: phylogenetic incongruence of core gene, gene duplication, gene localization in a BGC. Shown are the gene numbers and the percentage for each criterium. Some of the genes fulfill several criteria; therefore, the total numbers and percentage of the genes exceeds 790 and 100%, respectively.

	**No. of genes**	**Percentages of genes**
**Phylogeny**	612	77 %
**Duplication**	338	43 %
**BGC**	205	26 %

Supplementary Table S4: Occurrence of ‘resistance indicator’ (RI) genes within the 406 analyzed genome-sequenced strains from the DSMZ strain collection using the Ψ-footprinting method. Numbers and the percentages of all RI genes are shown.

**No. of RI gene(s)**	**No. of strains**	**Percentages of strains**
0	265	65%
1	36	9%
2	55	14%
3	22	6%
4	18	4%
5	9	2%
6	0	0%
7	1	0.2%

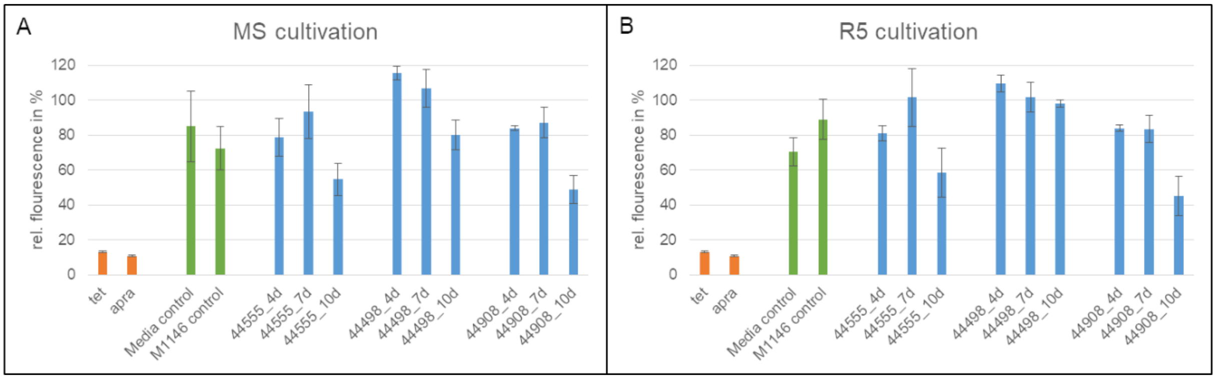

Supplementary Figure S4: *In vitro* transcription/translation assay performed with culture extracts in MS (A) and R5 (B) media from 4, 7, and 10 days. PSI antibiotics, tet_{15} and $apra_{50}$ = positive control (orange), medium extracts, extracts of M1146 = negative control (green), and extracts of DSM cultures (blue). Results are shown for samples, which did not lead to an inhibition of the *iv*TT assay. Measurements have been performed in triplicate using the same preparation of S12 extract.

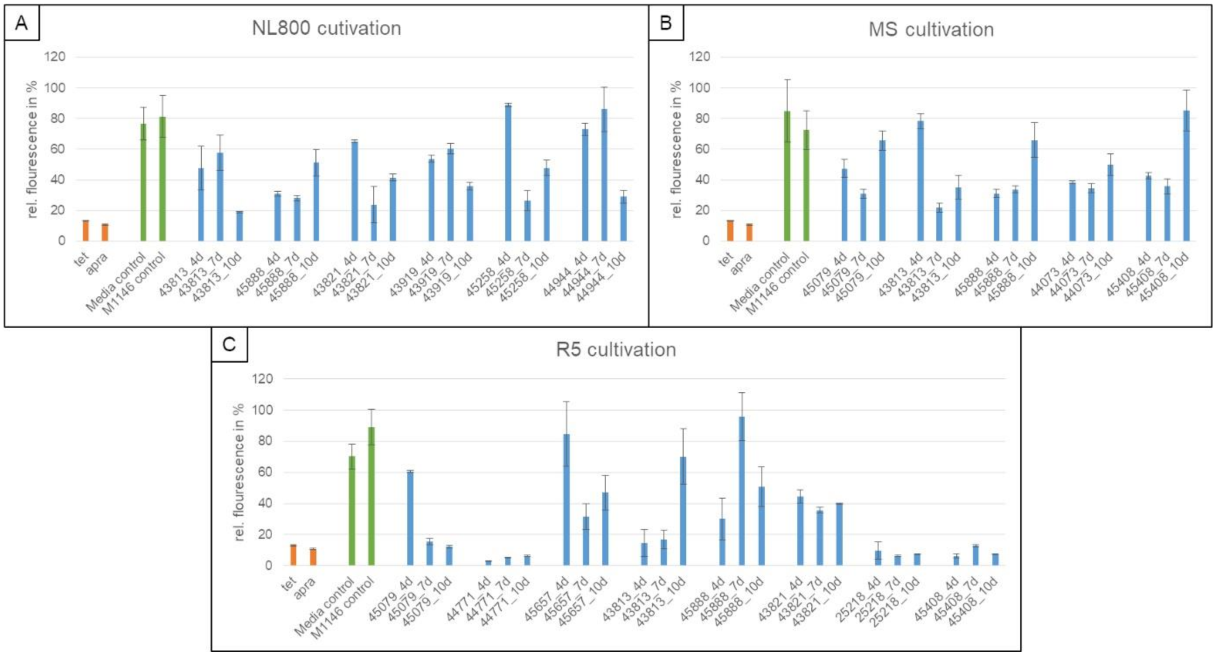

Supplementary Figure S5: *In vitro* transcription/translation assay performed with culture extracts in NL800 (A), MS (B), and R5 (C) media from days 4, 7, and 10. Only strains are shown, which were able to grow in the respective media. PSI antibiotics, tet_{15} and $apra_{50}$ = positive control (orange), medium extracts, extracts of M1146 = negative control (green), and extracts of DSM cultures (blue). Measurements have been performed in triplicate using the same preparation of S12 extract.

Supplementary Figure S5 - Additional information: Description of additional *in vitro* transcription/translation (*iv*TT) assay results:

*Iv*TT assays with extract samples of strain DSM 44944 incubated for 10 days in NL800 medium resulted in inhibition of GFP expression with values of 29% maximal fluorescence (Figure 6, Supplementary Figure S5). Extract samples of strain DSM 45888, DSM 45258, and DSM 43821 grown for 7 days in NL800 showed similar values (28%, 26%, and 24%, respectively) (Figure 6, Supplementary Figure S5). Inhibition of the *iv*TT assay suggests that DSM 44944, DSM 45888, DSM 45258, and DSM 43821 produce a PSI when grown in NL800, whereby the time point for best production varies.

*Iv*TT assays with culture extracts of DMS 44073 and DMS 45079 grown for 7 days in MS medium resulted in the inhibition of GFP expression with fluorescence values of 35% and 31%, respectively, indicating the presence of a PSI in both extracts (Figure 6, Supplementary Figure S5). Culture extracts of DMS 45657 grown for 7 days in R5 medium yielded similar inhibition results with maximal fluorescence values of 32%.

*Iv*TT assays with culture extracts of DSM 43813, DSM 45079, DSM 45408, DSM 25218, and DSM 44771 grown in R5 resulted in the lowest fluorescence values (<20% maximal fluorescence), which indicates strong inhibition of GFP expression (Figure 6, Supplementary Figure S5). Extracts from strains DSM 43813 and DSM 45079 grown for 7 and 10 days showed an inhibiting effect on GFP expression with fluorescence values of 17% and 12%, respectively.

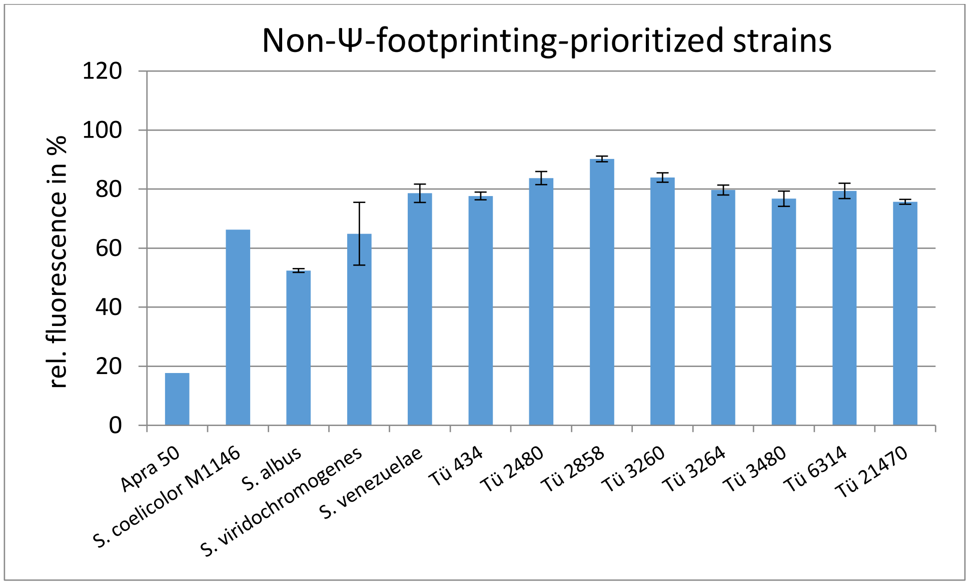

Supplementary Figure S6: *In vitro* transcription/translation assay performed with extract samples from strains of the Tübingen strain collection, which have not been prioritized with the Ψ-footprinting method. Apra50 = positive control. Extracts have been generated from strains of the Tübingen strain collection cultivated in R5 medium for 7 days. Measurements have been performed in triplicate using the same preparation of S12 extract.

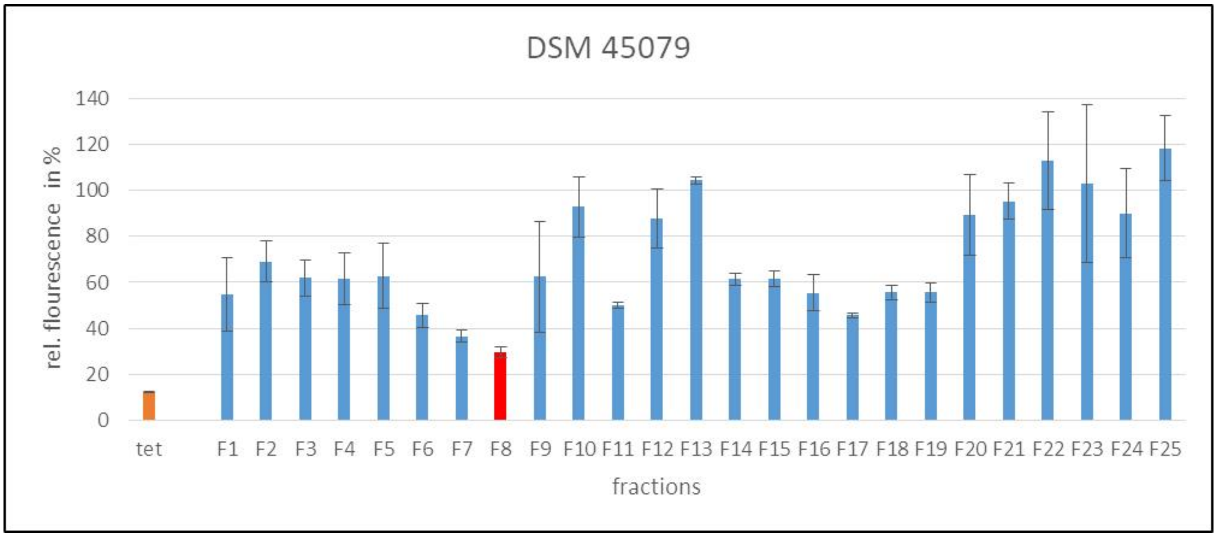

Supplementary Figure S7: *In vitro* transcription/translation assay performed with R5 culture extract of DSM 45079 from day 10. Displayed are the fractions generated by semi-preparative HPLC (blue). PSI antibiotic tet_{15} = positive control (orange). Fraction having the greatest inhibition is shown in red. Measurements have been performed in triplicate using the same preparation of S12 extract.

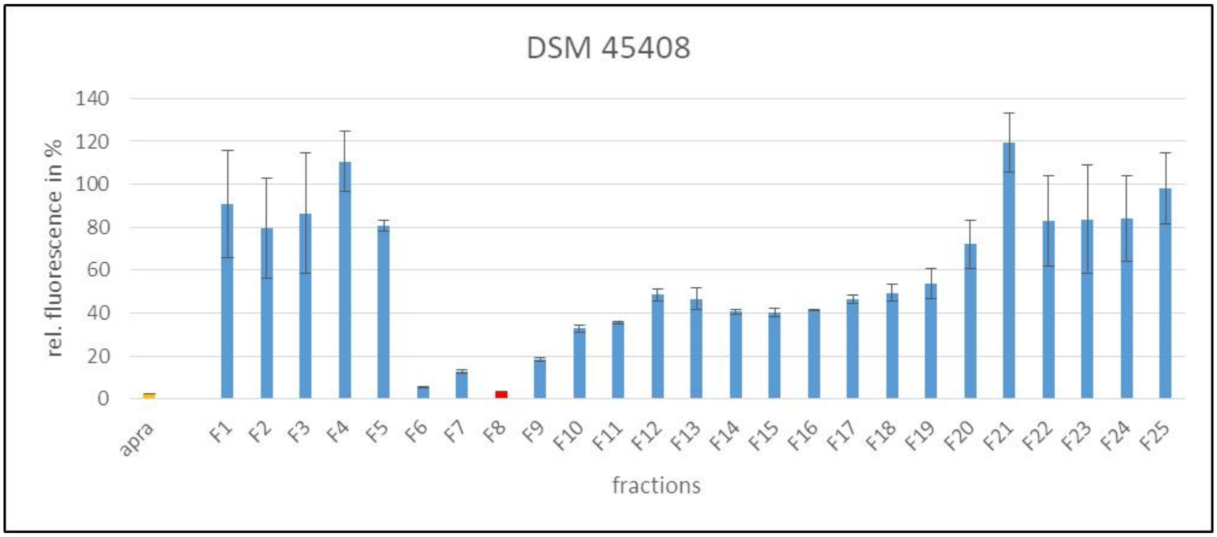

Supplementary Figure S8: *In vitro* transcription/translation assay performed with R5 culture extract of DSM 45408 from day 10. Displayed are the fractions generated by semi-preparative HPLC (blue). PSI antibiotic $apra_{50}$ = positive control (orange). Fraction having the greatest inhibition is shown in red Measurements have been performed in triplicate using the same preparation of S12 extract.

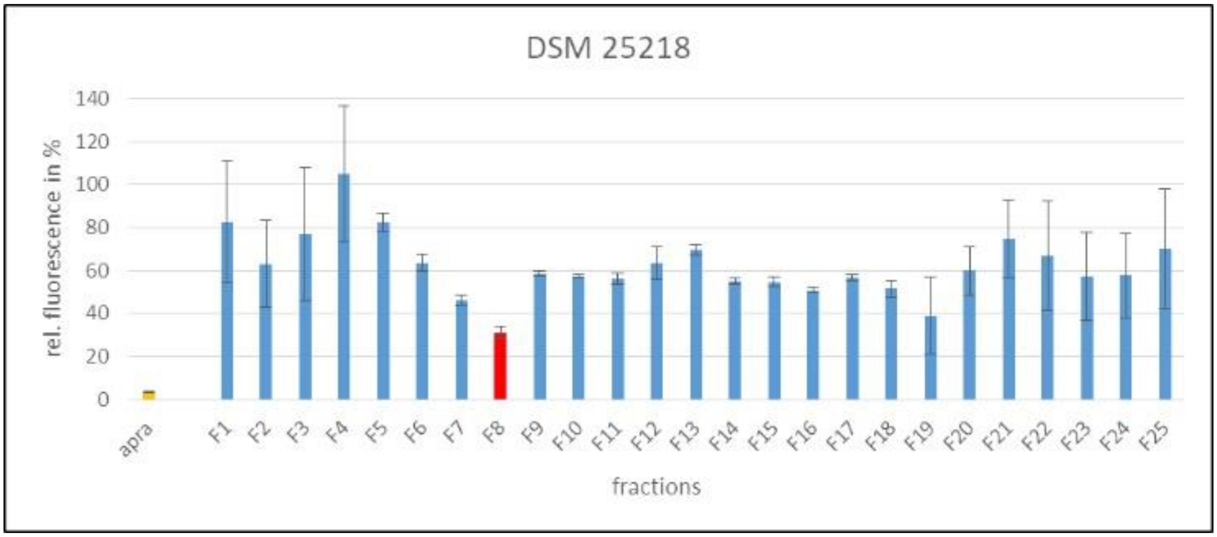

Supplementary Figure S9: *In vitro* transcription/translation assay performed with R5 culture extract of DSM 25218 from day 7. Displayed are the fractions generated by semi-preparative HPLC (blue). PSI antibiotic $apra_{50}$ = positive control (orange). Fraction having the greatest inhibition is shown in red Measurements have been performed in triplicate using the same preparation of S12 extract.

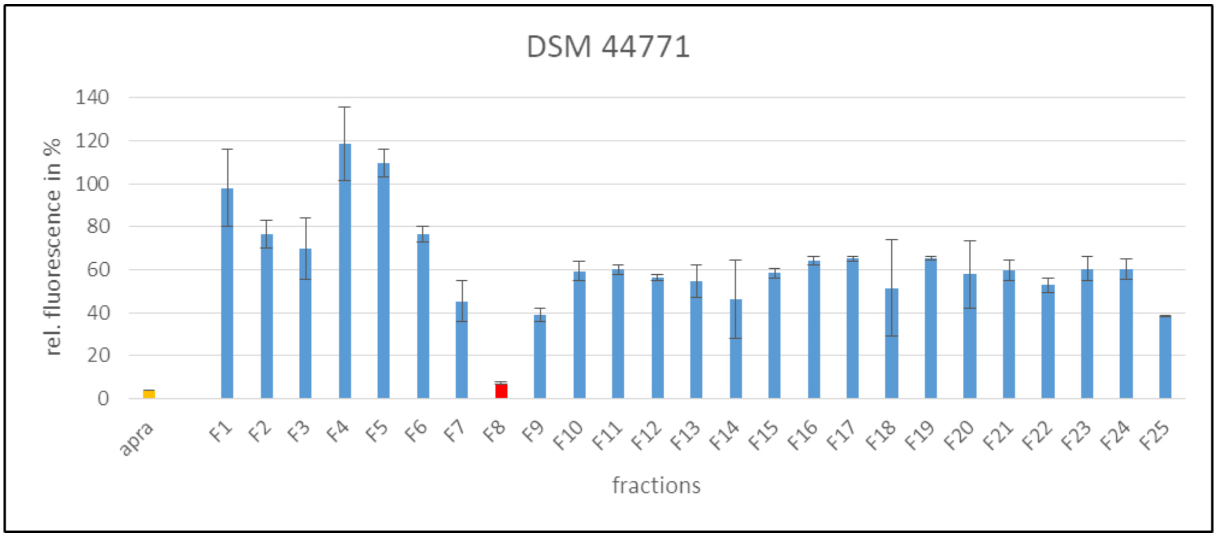

Supplementary Figure S10: *In vitro* transcription/translation assay performed with R5 culture extract of DSM 44771 from day 4. Displayed are the fractions generated by semi-preparative HPLC (blue). PSI antibiotic $apra_{50}$ = positive control (orange). Fraction having the greatest inhibition is shown in red Measurements have been performed in triplicate using the same preparation of S12 extract.

HRMS analysis

High resolution mass spectrometry was done with a Bruker Elute UHPLC 1300 coupled with a QTOF (Impact II, Bruker). 2 µl extract were injected on a Kinetex 100 C18 column (50 x 2.1 mm, 1.7 µm, Phenomenex) and eluted by a linear gradient from 90 % solvent A (water with 0.1 % formic acid) to 100 % solvent B (acetonitrile with 0.1 % formic acid) over 10 minutes with a flow rate of 0.5 ml/min. QTOF parameters were as follows: capillary voltage 4500 V, nebulizer 2 bar, drying gas 8 l/min, dry heater 220 °C. Data evaluation was conducted with Bruker Compass DataAnalysis 5.2.

Amicoumacin A

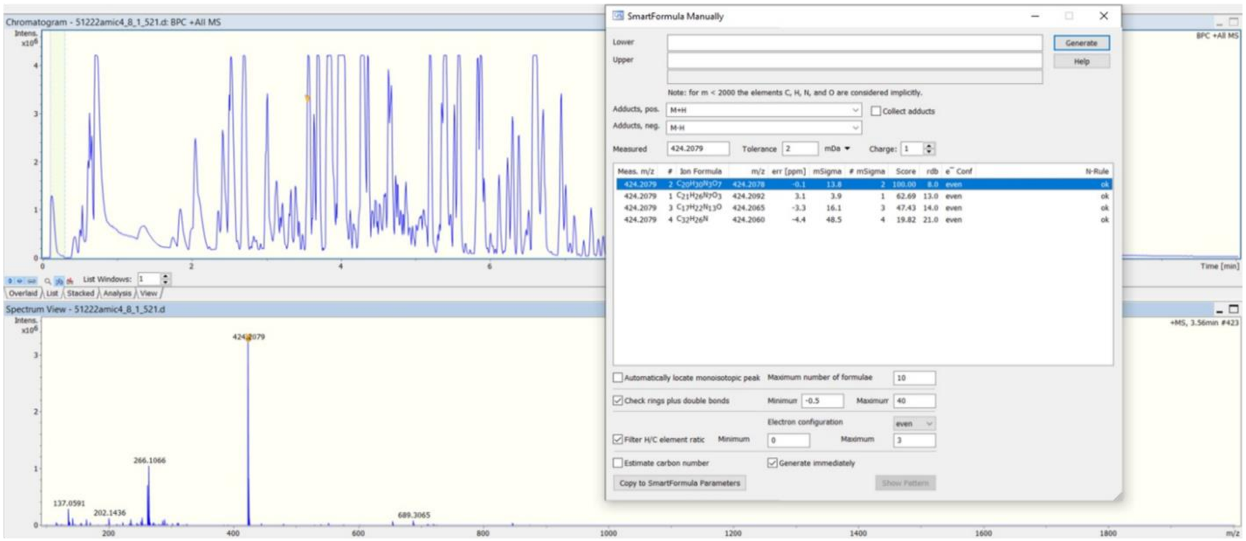

Amicoumacin B

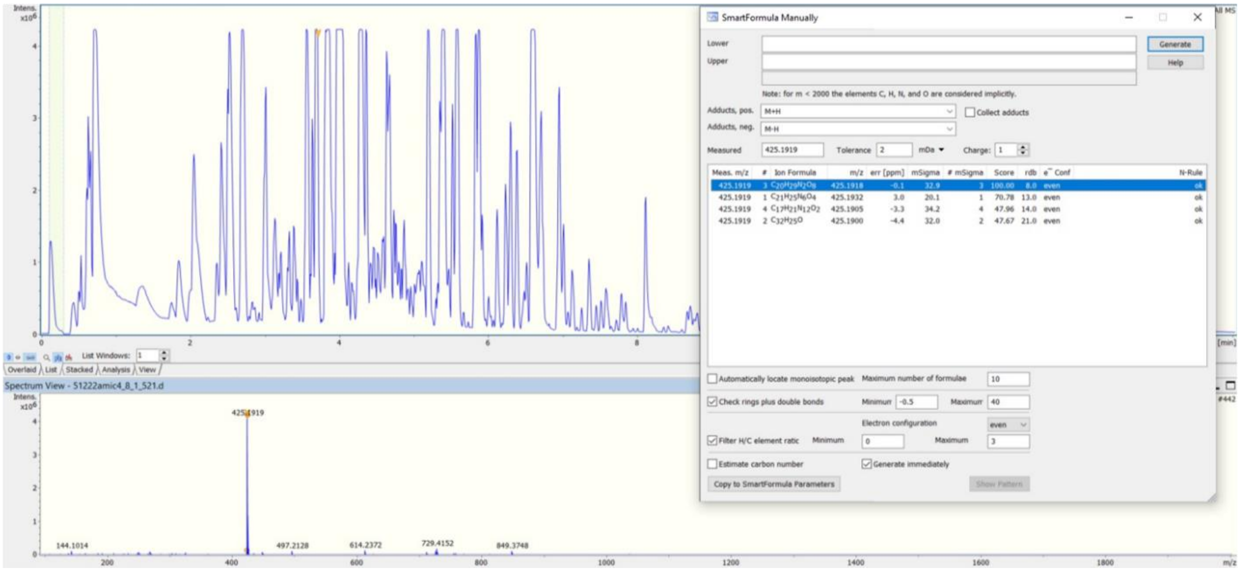

Supplementary Figure S11: HRMS data of sample F8 from DSM 45408. Positive extracted ion chromatograms (EICs) and MS^1 of amicoumacin A (above) and B (below). Expected mass for amicoumacin A ($C_{20}H_{30}N_3O_7$) is 424.2078 and for amicoumacin B ($C_{20}H_{29}N_2O_8$) 425.1918, error rates are -0.1 ppm.

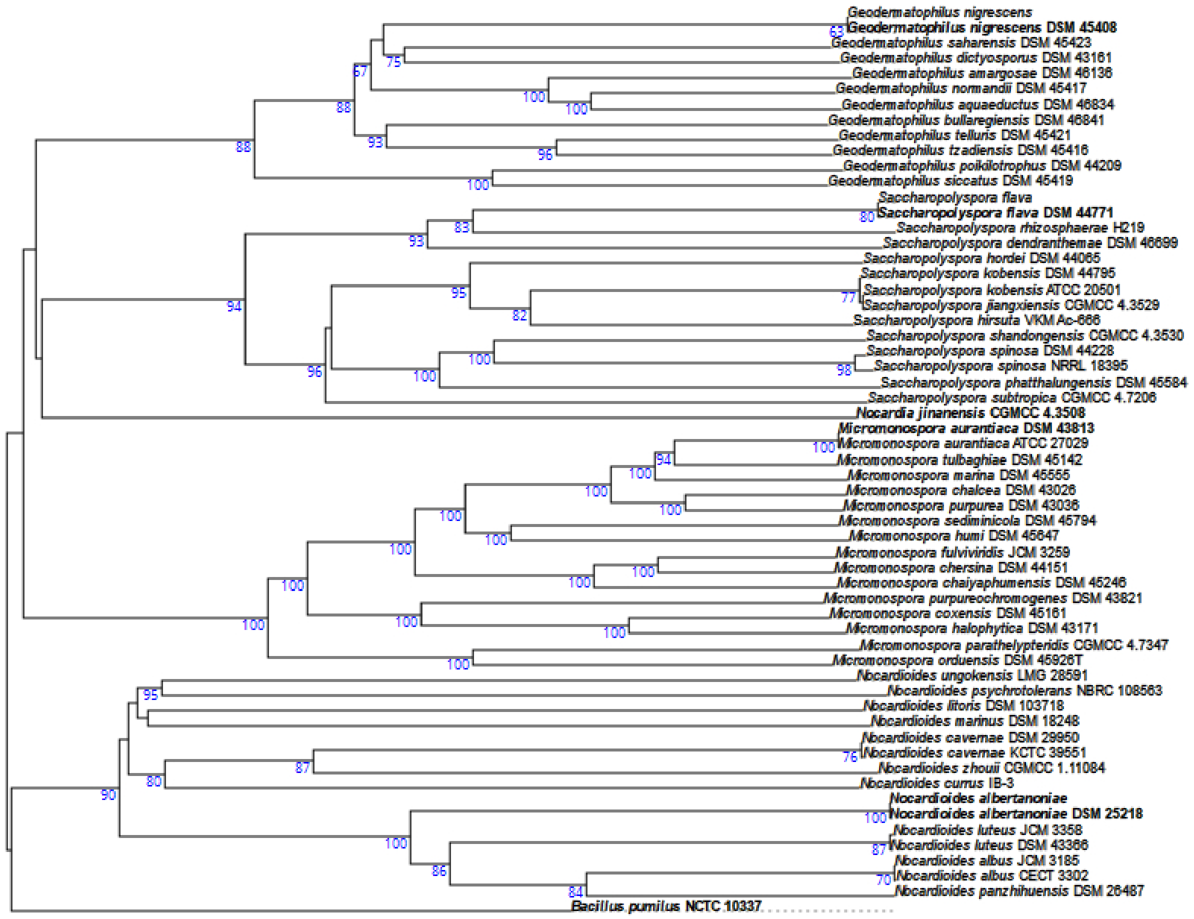

Supplementary Figure S12: Whole-genome sequence-based phylogenetic tree generated with the TYGS web server for the amicoumacin B producer strains DSM 44771, DSM 43813, DSM 25218, DSM 45408, and closely related type strains. The known amicoumacin producers *Bacillus pumulus* and *Nocardia jinanensis* were added manually to the reference strains. All amicoumacin producer strains are marked in bold. Tree inferred with FastME from GBDP distances calculated from genome sequences. The branch lengths are scaled in terms of GBDP distance formula d_5. The numbers above branches are GBDP pseudo-bootstrap support values >62% from 100 replications, with an average branch support of 85.3%.

References

1. Kieser,T., Bibb,M.J., Buttner,M.J., Chater,K.F., Hopwood,D.A. and John Innes Foundation (2000) Practical *Streptomyces* Genetics. Engl. 3. John Innes Found. Norwich.

2. Lau,R.C.M. and Rinehart,K.L. (1994) Berninamycins B,C, and D, minor metabolites from *Streptomyces bernensis*. *J. Antibiot. (Tokyo).*, **47**, 1466–1472.

3. Taylor & Francis Group (2020) Dictionary of Natural Products 30.1. *CRC Press*.

4. Ninomiya,A. and Kodani,S. (2013) Isolation of New Thiopeptide Berninamycin E from *Streptomyces atroolivaceus*. *Asian J. Chem.*, **25**, 490–492.

5. Wex,K.W., Saur,J.S., Handel,F., Ortlieb,N., Mokeev,V., Kulik,A., Niedermeyer,T.H.J., Mast,Y., Grond,S., Berscheid,A., *et al.* (2021) Bioreporters for direct mode of action-informed screening of antibiotic producer strains. *Cell Chem. Biol.*, **28**, 1242-1252.e4.

6. Malcolmson,S.J., Young,T.S., Ruby,J.G., Skewes-Cox,P. and Walsh,C.T. (2013) The posttranslational modification cascade to the thiopeptide berninamycin generates linear forms and altered macrocyclic scaffolds. *Proc. Natl. Acad. Sci. U. S. A.*, **110**, 8483–8.

7. Ninomiya,A. and Kodani,S. (2013) Isolation and identification of berninamycin A from *Streptomyces atroolivaceus*. Trade Science Inc.
